# Malware detection in IoT networks with CNNs and integrated feature engineering

**DOI:** 10.1038/s41598-026-47389-7

**Published:** 2026-04-20

**Authors:** Mahmoud Khaled Abd-Ellah, Nayera A. Alsayed, Osama M. Elkomy, Walaa M. EL-Hady

**Affiliations:** 1 Faculty of Artificial Intelligence, Egyptian Russian University, Cairo, 11829 Egypt; 2 Department of Information Technology, Faculty of Computers and Informatics, Zagazig University, Zagazig, Egypt

**Keywords:** Deep learning, Malware detection, IoT security, Network traffic, Convolutional neural networks (CNN), Feature extraction, Computational biology and bioinformatics, Engineering, Mathematics and computing

## Abstract

Malware poses a significant threat to Internet of Things (IoT) systems, with evolving stealth techniques challenging traditional detection methods. Effective identification of complex and diverse malware patterns requires advanced analytical approaches. We propose a deep convolutional neural network (CNN) framework integrated with comprehensive preprocessing pipelines, including normalization, encoding, and feature engineering techniques applied to structured network traffic data. Categorical traffic attributes were transformed into numerical representations using methods such as Bag of Words, TF-IDF, Word2Vec, and PCA to generate fixed-length feature vectors compatible with CNN architectures. Five CNN architectures were evaluated, with the best models achieving 100% accuracy and perfect AUC scores, demonstrating robust classification capabilities. These results indicate that combining deep learning with sophisticated preprocessing and feature engineering can significantly improve malware detection performance in IoT environments. This approach offers a promising direction for developing adaptive and reliable security solutions against emerging cyber threats in connected systems.

## Introduction

 Malware continues to evolve rapidly despite advances in cybersecurity methods, making it increasingly difficult to detect and understand due to its growing complexity and stealth. Cybercriminals employ sophisticated counter-analysis techniques, such as code encapsulation and compression, to conceal attacks and hinder analysis^1^. Additionally, the expanding diversity of digital environments, communication platforms, and computing technologies has enabled malware to appear in numerous forms. Consequently, malware analysis has become more challenging and necessitates the adoption of advanced solutions to address these complex threats^2^.

Current cybersecurity efforts employ scanning systems to identify abnormal or risky activities, utilizing various analytical approaches such as statistical, content-based, dynamic, and static analysis^3^. However, attackers continually develop new malware specifically designed to evade traditional antivirus software, exploiting knowledge of these defensive strategies. Malware refers to software intended to monitor computer activity, gain unauthorized access to systems, or collect sensitive information^4^.

Malware is any program intentionally designed to perform harmful actions on computers, smartphones, networks, or other targeted devices. Various types of malware exist, including ransomware, rootkits, worms, Trojan horses, and viruses. Each type is engineered to impact victims in specific ways, such as damaging targeted systems, enabling remote code execution, or stealing sensitive data^5,6^. Detecting malware is crucial for protecting users and organizations from these threats. Initially, signature-based detection was used, but it is ineffective against new or sophisticated malware. Consequently, researchers have developed more advanced methods, such as behavior-based, heuristic, and pattern-based detection. As threats continue to evolve, data mining and machine learning techniques have become increasingly important for enhancing malware detection and improving protection systems^5^.

Given the dynamic and increasingly complex nature of malware patterns, artificial intelligence and machine learning have proven highly effective for malware detection and classification. Various machine learning classifiers, such as Logistic Regression, Naive Bayes, and K-Nearest Neighbors, have been successfully applied to improve detection efficiency and accuracy^7^. Additionally, techniques like Support Vector Machines, Convolutional Neural Networks, Random Forests, and Hidden Markov Models have been employed in malware detection and classification tasks^8^. However, as ML-based systems advance, attackers also adapt their strategies, presenting new challenges^9,10^. Furthermore, the lack of transparency in traditional machine learning models can limit their applicability in certain contexts^9^.

Detecting malware is a significant security issue that is closely related to businesses’ legal, reputational, and financial worries^11^. Prior research has explored both ML and DL methods to tackle malware detection challenges^12^. While some studies focused on traditional ML algorithms, others investigated the capabilities of DL models in identifying malicious behavior^13–15^.But many issues with malware detection can be resolved with deep learning to create and improve detection algorithms^11^. DL techniques are very important for finding malware because they can change to new threats and pull out complicated characteristics from unprocessed data. Convolutional and recurrent neural networks, such as CNNs and LSTMs, have the ability to extract patterns from both static attributes and dynamic behaviors of malware. This dual capability supports a more complete understanding of malicious activity. Moreover, deep learning reduces reliance on manually updated rules, which enhances scalability and operational efficiency in real-world environments^16,17^.

Although numerous studies have applied deep learning algorithms to malware detection, many still face significant limitations that affect model effectiveness. For example, some studies have relied on small datasets, which hinders the models’ ability to generalize to real-world scenarios^18–21^. Additionally, many works lack the integration of advanced deep learning architectures that are better suited for capturing complex malware behaviors^2^. Existing research often uses limited datasets that do not cover the full spectrum of IoT malware types^22^, and some employ deep learning models with suboptimal performance due to inadequate preprocessing or feature extraction steps, as demonstrated in^23^.

The primary objective of this research is to develop a robust malware detection framework using convolutional neural networks (CNNs) to address the limitations identified in previous studies. The proposed approach incorporates a range of preprocessing and feature extraction techniques, including normalization, TF-IDF, Bag of Words, Word2Vec, PCA, and RFE to enhance data quality and representation. By experimenting with different CNN architectures and configurations, the study aims to improve detection accuracy and ensure adaptability to diverse malware behaviors.

The remainder of the paper is organized as follows: Sect.  2 presents the literature review, Sect.  3 describes the proposed methodology, and Sect.  4 outlines the experimental environment. Sections  5 and 6 present the results and discussion, respectively, followed by the conclusion in the final section.

## Literature review

As the malware analysis techniques advance, security challenges are increasing as a result of the increasing utilization of intelligent technologies and the rise of malware attacks. This is due to the wide variety of connected devices, which vary in their capabilities in terms of hardware, software, and network architecture, making securing IoT environments more complex^24^.

Recent literature reviews in the field of IoT malware detection have highlighted both established and emerging challenges, as well as areas requiring further research and development^25,26^. These reviews contribute to building a strong knowledge base by synthesizing research findings and identifying trends relevant to the advancement of malware detection techniques.

IoT is rapidly expanding globally; however, securing it remains a challenge. Machine learning techniques have proven effective in detecting malware on these devices, which often lack constant monitoring^27^. Models and methods for deep learning have become an essential part of improving malware analysis in IoT environments, thanks to being capable of handling enormous volumes of data through a multi-layered architecture, enabling higher levels of accuracy and efficiency in threat detection^24^.

This part aims to review recent studies on the application of machine learning and deep learning techniques for malware detection. The primary objective will be to examine the different approaches that the research has employed, including preprocessing, feature selection, feature extraction, and classifier design. Each study’s advantages and disadvantages will be compared as well, highlighting any knowledge gaps and prospective challenges in this field.

In^28^, the authors proposed a deep learning–based intrusion detection framework for IoT networks using the CIC IoT-DIAD 2024 dataset. The data were preprocessed using StandardScaler and label encoding, while feature extraction was automatically performed through deep learning models. Several classifiers were evaluated, including 1D CNN, LSTM, RNN, and MLP. The 1D CNN achieved the best performance with an accuracy and F1-score of 99.53%. Although deep models such as LSTM and RNN suffer from high training cost and vanishing gradient issues, the 1D CNN demonstrated superior accuracy with low false alarms and relatively low computational complexity, making it suitable for IoT intrusion detection.

In^29^, a hybrid deep learning model combining an LSTM-Autoencoder with a Multilayer Perceptron (MLP) was proposed for IoT detection. The model employed data normalization and sequential encoding for preprocessing. Experimental results on the N-BaIoT2018 dataset showed high performance, achieving an accuracy of 99.77%, an F1-score of 99.87%, a precision of 99.92%, and a recall of 99.83%. Additionally, the model demonstrated strong generalization when evaluated on the UNSW-NB15 dataset, attaining an accuracy of 99.67% and an F1-score of 99.70%. Despite its high detection capability, the approach relies on a centralized server, which may introduce scalability and security limitations.

In^30^, an RNN-based approach was proposed for detecting malicious behavior in IoT applications using a dataset consisting of 281 malicious and 270 benign software samples. The proposed model achieved an accuracy of 98.18%. However, the small size of the dataset represents a major limitation, which affects the generalization ability of the model when applied to large-scale or real-world IoT environments.

The authors used CUDA-Accelerated Hybrid CNN-DNN to detect IoT network attacks in^31^. They employed CNNs for feature extraction and deep neural networks for detection and classification on the kitune dataset. Their model had 98.41% precision and 98.56% recall.

In^32^, logistic regression and ResNet-18 models were used in a machine learning study to detect network threats. The data was processed into network flows, features were identified and collected .pcap format. The top 15 features were selected from a variety of datasets and split into two groups. The ResNetDDoS-1 model exceeded the others with 98.70% accuracy, 97.53% precision, 97.96% recall, and 97.74% F1-score. The study suffers from several limitations, such as the study mostly concentrating on particular kinds of scanning and DDoS attacks, which does not cover all possible risks, and additional fine-tuning and validation on a wider range of datasets may be necessary to improve the models’ resilience.

The authors of^33^ employed machine learning methods and an ensemble architecture to identify malware in network data. They used the AdaBoost technique to combine Artificial Neural Networks (ANN), Naive Bayes (NB), and Decision Trees (DT) into an ensemble framework. The UNSW-NB15 dataset was used for testing, and the NIMS Botnet Dataset and simulated IoT sensor network traffic were used to evaluate how well the framework worked. By using UNSW-NB15 dataset, the AdaBoost ensemble framework achieved an accuracy of 99.54%, DT achieved an accuracy of 95.32%, ANN achieved an accuracy of 92.61%, and NB achieved an accuracy of 91.17%; and by using the NIMS Dataset, the AdaBoost ensemble framework achieved an accuracy of 98.29%, DT achieved an accuracy of 96.10%, ANN achieved an accuracy of 94.22%, and NB achieved an accuracy of 88.28%, but it had some problems, like how hard current attacks are and how much they depend on feature quality generalizability.

In^23^, researchers developed a hybrid method using CNNs and LSTMs to detect botnet attacks on IoT devices. The N-BaIoT dataset, which includes data from nine IoT devices, was used to gather data from BASHLITE and Mirai attacks.The CNN-LSTM hybrid model demonstrated exceptional performance across various devices, with the Danmini doorbell, Ennio doorbell, thermostat, baby monitor, and security cameras all achieving high accuracy rates, with the Danmini doorbell achieving 90.88%, Ennio at 88.61%, the thermostat at 88.53%, and the baby monitor at 91.58%. However, the study identified drawbacks, such as the challenge of detecting botnet assaults that closely imitate legitimate traffic, leading to low detection rates and recall values. Additionally, the CNN-LSTM model requires significant computing power for training.

- M. Amin et al. (2022) developed advanced ways to find harmful Android apps by using DL and ML models in^34^. They used one-hot encoding and bytecode extraction from APK files as preprocessing. The researchers used the Chars2Vec method to select and extract features. The two deep learning models they assessed were the LSTM and the Fully Connected Neural Network (FCN). The FCN model achieved 98.9%, while the LSTM model achieved 98.9%. The inefficiency of some feature detectors in managing code obfuscation techniques and poor performance when dynamic code loading took place during runtime were among the challenges the study faced.
- B. Taşcı et al. (2024) used ML algorithms in^35^. They used the CIC IoT 2023 dataset and compared the performance of the model with that of other models, such as Naive Bayes, KNN, SVM, decision trees, and1D CNN. The 1D CNN model outperformed the rest, attaining an accuracy of 98.36%. However, there are some limitations, including the necessity for validation on a large dataset and additional assessment of the model’s efficiency and usefulness in real-time applications, especially on low-resource devices.
- M. Azeem et al. (2024) used ML techniques to detect malware using the UNSW-NB15 dataset in^2^. They employed Information Gain and Gain Ratio, as well as Bag-of-Words, Word2Vec, and TF-IDF for feature extraction. They used multiple classifiers to train the model. The ET classifier showed the highest accuracy at 99.98%. However, the study’s limitations encompass its dependence on the UNSW-NB15 dataset, its potential incapacity to fully capture complex relationships between categorical variables, and the absence of deep learning techniques.

In^36^, the authors created an ensemble classifier that uses models from LSTM, RNN, and CNN to detect malicious activity in IoT scenarios. Training uses 10% of the BoT-IoT dataset, which includes five output classes, 46 features, and 3,000 keylogging attack recordings. They used CSE-CIC-ID2018 for validation. With an accuracy of 97.67%, precision of 97.72%, recall of 97.68%, and F1-score of 97.70%, the ensemble model outperforms individual models. However, the study has drawbacks, including high processing demands, data limitations due to a lack of training data for keylogging assaults, and complexity due to the need for specialized knowledge.

The study in^37^ proposed a framework to detect malware. The authors used Radare2 to extract control flow graphs and calculate graphical metrics for feature selection during the preprocessing .They employed CNNs, Random Forests (RFs), Support Vector Machines (SVMs), and Logistic Regression (LR) models and evaluated the framework on the dataset that contains 2,962 malware samples, randomly selected from CyberIOCs. The CNN model showed the highest accuracy at 99.66%.The study lists a number of limitations, including the potential for static analysis to overlook malware’s dynamic activity and the ability of malware to employ camouflage techniques that diminish the effectiveness of program-level detection.

In^38^, the authors introduced the ACLR model to detect network-origin attacks. The model underwent the application of four distinct DL architectures: the Artificial Neural Network (ANN), CNN, LSTM, and RNN. They used the UNSW-NB15 dataset, which included nine attack types and 82,332 network traffic records. The proposed ACLR model achieved an accuracy rate of 97.49%, a precision of 97.7%, a recall of 97.17%, and an F1-Score of 97.23%. The study has a number of limitations, including the fact that the performance depends on labeled data, which might not always be representative.

In^39^, the authors employed various ML techniques, including RF, XGBoost (XGB), DT, Gradient Boosting (GB), LR, SVM, and K-Nearest Neighbor (KNN). Malware samples obtained from sites such as VirusTotal, AnyRun, and PolySwarm were used to create the dataset. The results of the evaluation showed that the algorithms performed differently, with the Random Forest (RF) model recording an accuracy of 93.3% and the XGBoost (XGB) model obtaining an accuracy of 93.0%. Decision Tree (DT) obtained 90.9% accuracy, while Gradient Boosting (GB) achieved 90.0%. The K-Nearest Neighbor (KNN) and Support Vector Machine (SVM) models’ accuracy is not specified, while the Logistic Regression (LR) model’s accuracy was 86.7%.The study has some problems, such as ML models facing challenges due to insufficient training samples, especially for fileless malware, which may not accurately represent the malware types being detected.

In^40^, researchers have developed a ML-based method to detect malicious data in IoT networks. They used KNN, RF and Gaussian Naïve Bayes (GNB). The models were tested on a sample of simulated IoT traffic that included both malicious and benign traffic. The k-NN model had an accuracy of 94.44%, which was better. The RF model achieved an accuracy of 88.8%, while GNB had a poor accuracy of 77.78%. However, the study has limitations, including the incapacity to detect new malware that hasn’t been discovered yet and the lack of clarity in the data source documentation, which makes it difficult to verify or duplicate the findings.

In^41^, characteristics were selected and extracted from three main categories: a hybrid approach, fixed features like permissions and API summonses, and dynamic features like network activity and regime calls. To test the model, 6192 safe apps and 5560 malware samples were taken from the Google Play and Chinese app stores. The results showed that, with a 98% effectiveness rate in identifying dangerous programs, the NAIVE Bayes model was effective in identifying hazardous applications. But the study identified several problems that may undermine the effectiveness of the analysis, the most significant of which was the inability to retrieve certain features when using the Dex2jar tool to decompile APK files.

In^42^, this study developed a sophisticated engineering method that integrates variable automatic encryption (VAE) with classic automatic encryption (CAE). The authors used a deep neural network with batch normalization (DNN-BN) to improve detection accuracy and model stability. The dataset that was used, including 14,700 dynamic analytic operations features; was 70% for training and 30% for testing. According to the results, the model obtained an F1 score of 94.71% and an accuracy of 95.38%.However, the benefits of features and the complexities of preprocessing methods, which require a significant amount of computational power, present difficulties for the study.

In^43^, this study presented DWARF Mongoose Optimization with ML-Driven Ransomware Detection (DWOML-RWD) to find ransomware attacks. The authors used the Enhanced Krill Herd Optimization (EKHO) model and dynamic opposition-based learning (DOBL) for feature selection and used the Extreme Learning Machine (ELM) model to detect things and DWO Mongoose Optimization (DWO) to modify the ELM model’s parameters. They compared the method to Adaboost-M1, Bagging, RF, Rotation Forest (ROF), and DT to see how well it worked. The DWOML-RD model performed well, achieving 99.40% accuracy, 99.40% recall, and 99.40% F1score. The study’s limited database scope.

A comparative analysis of recent studies that have looked into various deep learning, machine learning, and hybrid approaches is provided in Table 1. The table highlights each study’s methodology, dataset, feature extraction approach, and reported performance metrics. This review aims to give readers a clear understanding of the various approaches used in IoT malware detection. These studies employ a variety of preprocessing techniques, feature engineering techniques, and detection models, each of which offers a unique perspective on the effectiveness of the current research trends.

**Table 1:** Comparative summary of recent approaches for IoT malware detection using various techniques.

RF	Method			Result %				Dataset	Limitation	Advantage
	Pre-processing	Feature Selection	Detection	Acc	F1-score	Per scion	Re-call			
^28^	StandardScaler	Automatic feature extraction via convolutional layers	1D CNN	99.53	99.53	99.53	99.53	CIC IoT-DIAD 2024	High memory and training costs, Vanishing gradient issues	Highest accuracy, low false alarms, low computational cost
			LSTM	99.52	99.52	--	--			
	Label encoding		RNN	99.25	99.25	--	--			
			MLP	98.78	98.78	--	--			
^29^	Data normalization	--	Hybrid LSTM-Autoencoder + MLP	99.77	99.87	99.92	99.83	N-BaIoT2018	Centralized server dependency	Strong generalization across datasets
	Sequential encoding			99.67	99.70	99.62	99.78	UNSW-NB15		
^30^	--	--	RNN	98.18	--	--	--	Dataset of IoT applications that includes 281 malicious and 270 benign pieces of software	Small size of dataset	--
^31^	--	CNN	CUDA- Hybrid Model (CNN-DNN)	98.41		98.41	98.56	collected from the kitune dataset	--	--
^32^	--	Logistic Regression (LR) algorithm	ResNetDDoS-1	98.99	99.04	98.91	99.18	CICIDS-19	The study primarily focused on specific types of scanning and DDoS attacks, which not encompass all potential threats. The models may require further tuning and validation on more diverse datasets to enhance robustness.	Effective feature selection techniques to improve model performance and reduce complexity.
				97.95	95.30	95.66	99.44	CICIDS-17		
				99.16	98.88	98.02	99.75	Bot-IoT		
				98.70	97.74	97.53	99.46	Average Result		
			ResNetDDoS-2	70.22	56.51	96.67	39.92	DDoSLab		
				78.41	40.86	63.01	31.03	CICIDS-17		
				68.58	27.60	93.56	12.57	Bot-IoT		
				72.40	30.13	84.41	27.84	Average Result		
			ResNetDDoS-3	64.15	9.79	70.81	5.26	DDoSLab		
				49.60	9.91	81.28	5.27	CICIDS-19		
				64.15	9.79	70.81	5.26	Bot-IoT		
				58.77	19.36	82.28	11.53	Average Result		
			ResNetDDoS-4	71.75	59.03	99.30	71.75	DDoSLab		
				47.53	0.44	70.31	0.63	CICIDS-19		
				78.14	1.29	56.90	6.05	CICIDS-17		
				65.81	20.25	75.50	14.29	Average Result		
^33^	--	--	DT	95.32	--	--	--	UNSW-NB15	Complexity of Modern Attacks Dependence on Feature Quality Generalizability	Enhanced Detection Performance: The use of the AdaBoost ensemble method leads to improved detection rates and reduced false positives.
				96.10	--	--	--	NIMS		
			NB	91.17	--	--	--	UNSW-NB15		
				88.28	--	--	--	NIMS		
			ANN	92.61	--	--	--	UNSW-NB15		
				94.22	--	--	--	NIMS		
			Ensemble Method: AdaBoost	99.54	98.93	--	--	UNSW-NB15		
				98.29	97.38	--	--	NIMS		
^34^	Bytecode	Chars2Vec	FCN	98.9	98.3	98.9	--	It contains 16,680 for benign dataset collection. and approximately 11,200 malicious apps	Feature detector may not be effective against code obfuscation. Ineffectiveness against dynamic code loading at runtime.	Handles large-scale datasets effectively without overfitting.
	One-hot encoding of opcodes.		LSTM	98.9	99.0	98.4	--			
^35^	Normalization	--	1D CNN	98.36	99.95	100	99.96	CIC IoT 2023 Dataset.	The model requires validation on a broader range of datasets to improve generalizability to various IoT environments. Although the model demonstrates low computational overhead, its performance and efficiency in real-time applications, especially on resource-constrained devices, need further evaluation.	Indicate the usefulness and efficiency of deep learning methods in enhancing IoT security.
	Encoding Categorical Variables									
^2^	Bag-of-Words	Entropy-based feature selection like (IG) and (GR)	ET	99.98	99.98	99.98	99.98	UNSWNB15	Single Dataset TFIDF does not capture complex relationships between categorical variables. Lack of Deep Learning	Identifies the best combinations by providing a thorough comparison of classifiers and encoding methods. Emphasizes dimensionality reduction to increase the accuracy and efficiency of the model.
			KNN	99.97	--	--	--			
	Word2Vec		RF	99.96	--	--	--			
			LR	99.96	--	--	--			
	TF-IDF		DT	99.95	--	--	--			
			MLP	99.96	--	--	--			
^36^	Transformation Normalization	--	Ensemble models classifier consisting of CNN, RNN, and LSTM	97.67	97.7	97.72	97.68	BoT-IoT	High processing demands, making it inappropriate for IoT devices with few resources. Data Limitations: Limited training data for keylogging attacks affect model robustness. Complexity: Implementation and maintenance require specialized expertise.	Low False Positive Rate: Reliable for real-world applications. Validation on Multiple Datasets: Demonstrated consistent performance across different datasets.
				95.03	95.07	95.09	95.05	CSE-CIC-ID2018		
^37^	CFG	--	CNN	99.66	--	--	--	2,962 malware samples, randomly selected from CyberIOCs	Malware is vulnerable to obfuscation techniques, affecting detection performance at the program level, and static analysis may overlook dynamic malware behavior.	High accuracy and low error rates using CNN for IoT malware detection and classification. Use of CFG-based features for distinguishing IoT and Android malware effectively
			SVM	97.65	--	--	--			
	Radare2		RF	98.48	--	--	--			
			LR	97.47	--	--	--			
^38^	--	--	ACLR	97.49	97.23	97.7	97.17	UNSW-NB15	The performance depends on labeled datasets, which might not always represent real-world scenarios.	Scalability: The proposed hybrid model adapts to various attack types effectively.
			ANN	75.68	75.59	78.17	75.68			
			CNN	94.40	94.39	94.44	94.40			
			LSTM	96.51	96.51	96.51	96.51			
			RNN	95.22	95.21	95.23	95.22			
^39^	--	--	RF	93.3	--	--	--	The dataset was downloaded from VirusTotal, AnyRun, and PolySwarm.	Accuracy has not been measured for some models. ML models face challenges due to insufficient training samples, especially for fileless malware, which may not accurately represent the malware types being detected.	--
			DT	90.9	--	--	--			
			SVM	--	--	--	--			
			LR	86.7	--	--	--			
			KNN	--	--	--	--			
			XGBoost	93.0	--	--	--			
			GB	90.0	--	--	--			
^40^	packet traffic capture	--	KNN	94.44	96	92	1	Simulated IoT traffic dataset	Difficulty in detecting undiscovered new malware, and lack of transparency in documenting the source of the data, making it difficult to verify or reproduce the results.	Scalable for large networks.
			RF	88.8	--	--	--			
			GNB	77.78	--	--	--			
^41^	Clustering algorithm	--	NB	98	98	98.2	--	Collected from Google Play and the Chinese app stores includes 6192 benign and 5560 malware	The study has problems with hiding features when decompiling APK files using Dex2jar.	--
^42^	CAE- VAE	--	DNN-BN	95.38	94.71	96.99	--	Included observations of system API packages	The preprocessing steps are complex and require significant computational resources.	DNN with batch normalization provided improved generalization, improving the model’s ability to adapt to new ransomware behaviors.
^43^	--	EKHO DOBL	DWOML-RWD Model	99.4	99.4	99.4	99.4	Consists of 840 samples with 420 goodware samples and 420 ransomware samples.	The database range is limited to 840 samples, which may restrict the ability to generalize in the real world and the variables of dynamic ransomware programs.	DWO algorithm enhances ELM performance through robust parameter tuning.

As seen in Table 1, the majority of current research relies on either a single deep learning model or a small number of feature extraction methods. Fewer studies, nevertheless, make an effort to compare or hybridize preprocessing techniques and various architectures. This discrepancy demonstrates the driving force behind our suggested work, which combines multiple feature engineering approaches and assesses four different deep learning models in uniform settings.

## Methodology

**Fig. 1:**
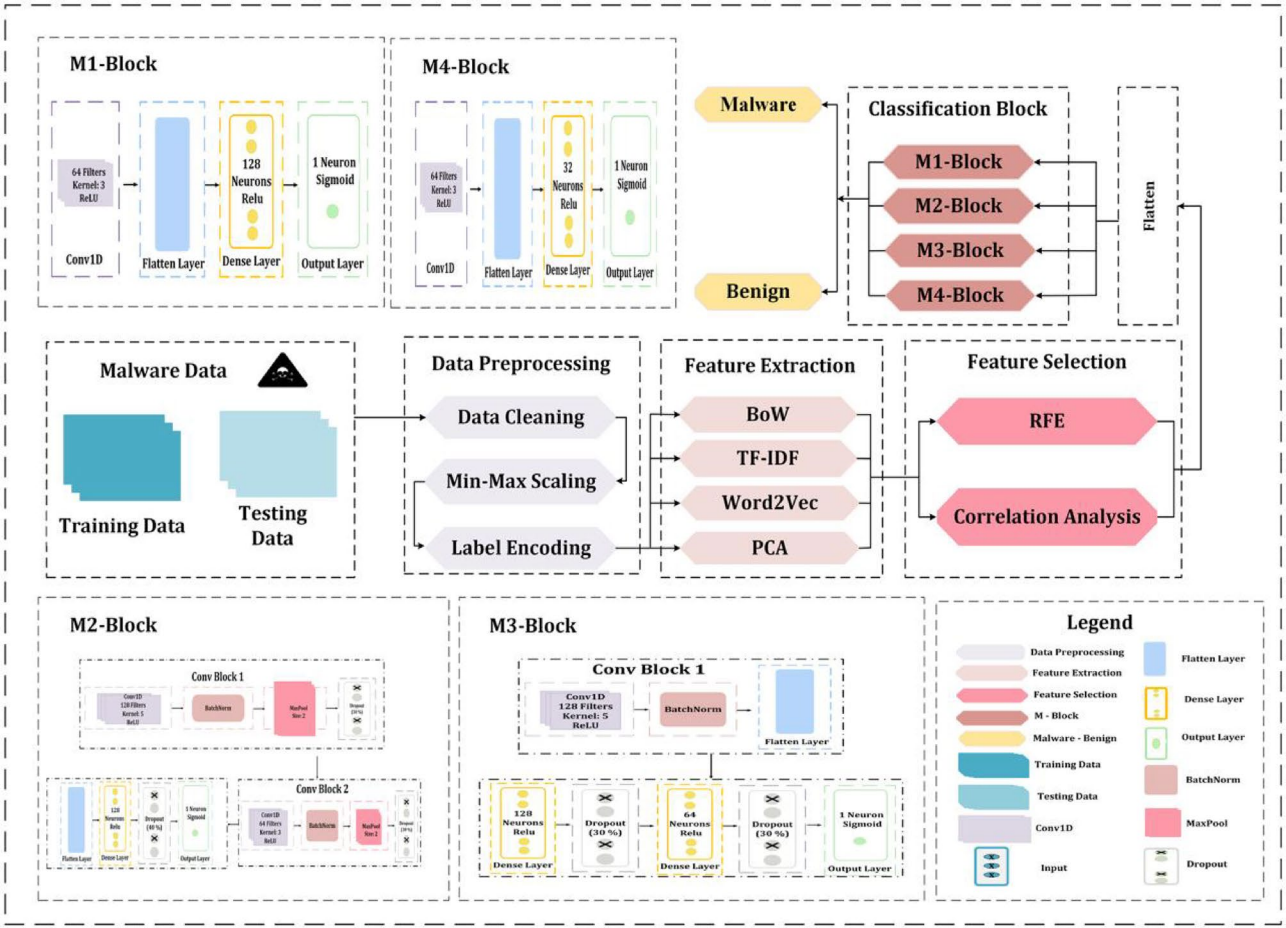
Proposed Methodology Framework for IoT Malware Detection using CNN models containing four M-Blocks.

In this part, we talk about the research’s methodological framework. It provides a thorough explanation of the dataset, feature engineering methods, preprocessing procedures, and suggested deep learning models for malware detection in IoT settings. The methodology is set up to guarantee conformity to the goals of the study. From data preparation to model evaluation, every step of the pipeline is carefully planned to improve detection performance and gauge how well different architectures work. We performed several experiments on a dataset to assess the efficacy of several preprocessing strategies, feature selection approaches, and CNN designs. Every experimental plan had a different mix of CNN model configuration, feature selection, and preprocessing. As shown in Fig. 1, the proposed approach consists of data preprocessing, feature extraction, feature selection, and input reshaping before training CNN models.

###  Data preprocessing

Every single operation that involves machine learning or deep learning must begin with data preprocessing as an important initial phase. The performance of the model is negatively impacted by other factors, like noise and missing values, inaccurate formulas, and irrelevant information that are frequently present in raw data. To improve the process of learning and attain better outcomes, the data must be cleaned, transformed, and normalized. The UNSW-NB15 dataset was preprocessed using a variety of methods in this work to enhance its quality and prepare it for CNN models.

The dataset was loaded into Pandas DataFrames. Before training a model, a lot of preprocessing is required because raw network traffic data often has mistakes like missing numbers, redundant data, and wrong entries. Initially, missing values ​​are identified across all features. Machine learning models suffer when NaN values are present. While this makes analysis easier, it can result in substantial data loss, particularly if missingness is common. If the missing data is not random, this can lead to biased results^44^. So we handled it by removing the rows that were affected. Duplicate records are then removed to avoid redundancy and make sure the model doesn’t pick up on repetitive patterns that could cause bias. Furthermore, specific numeric attributes, such as sbytes (source bytes), dur (duration), and dbytes (destination bytes), are analyzed for negative values to remove these values. Due to anomalous or damaged and lost data can cause incorrect model training and poor detection performance^45^.

####  Normalization

Numerical input is required by the majority of algorithms in deep learning and machine learning models. To prepare the data for model training, categorical textual features must be converted to numerical values. This numerical feature set in the dataset includes several significant parameters for network traffic analysis, including source packets (spkts), duration (dur), source bytes (sbytes), destination packets (dpkts), and destination bytes (dbytes). The characteristics mentioned above are essential for describing the general patterns of network traffic, which enhances analysis and detection accuracy.

In order to make them numerical representations that a deep learning model can understand, categorical attributes like attack_cat, state, protocol, and network service need to be encoded. To facilitate this, all unique values in these categorical columns are examined, and inconsistencies such as unknown entries (“-“, “?”, “unknown”) are identified and replaced with NaN and subsequently removed.

#### Min-max scaling

The normalization process applying the MinMaxScaler through Scikit-learn was used for scaling every number to a range of 0 to 1. This method makes deep learning models more stable and helps them converge^46^. Moreover, missing values were handled by replacing them with zeros, ensuring data completeness and preventing potential inconsistencies during model training. Equation (1) provides the mathematical expression for Min-Max Scaling^47^

1 $$T_{{scaled}} = \frac{{T - ~T\min }}{{T\max - T\min }}$$

Where T is the original data point, T _scaled_ is the scaled data point, T_min_ is the minimum value in the dataset, and T_max_ is the maximum value in the dataset.

####  Label encoding

Label encoding is a widely utilized technique for converting categorical variables into numerical values ​​by allocating a distinct number to each category, which involves giving every category with an integer beginning at )0 to *N* − 1^48^. We employed categorical encoding using the Label Encoding technique, where every category is given its own distinct numerical designation, allowing the models to process these features effectively. The mathematical representation of Label Encoding can be expressed as follows in Eq. (2)

2 $$L = E\left( {y_{i} } \right)$$

Where the element of the categorical feature is denoted by the letter yi in this context, the integer L is the one that is assigned to this particular category. L is a member of the set of integers {0, 1, 2, …, *n* − 1, where n represents the total number of distinct categories being considered.

These preprocessing methods ensure that the input data is clean, consistent, and properly scaled which increases the model’s ability to recognize patterns, decreases the risk of overfitting, and improves accuracy and performance in all areas.

### Feature extraction

The feature selection step follows preprocessing and is an essential part of any deep learning or machine learning workflow, especially when dealing with complex datasets like network traffic data. It is a technique used to eliminate features from the dataset that don’t address or aid in resolving the issue^45^. Its primary goal is to transform raw input data into meaningful representations that highlight the most important patterns and characteristics relevant for model training. Effective feature extraction enhances model performance, reduces computational complexity, and helps avoid overfitting.

In this study, multiple feature extraction techniques were applied to both numerical and textual features in the UNSW-NB15 dataset. These techniques aimed to capture the essential information from the data and generate feature vectors suitable for deep learning models. The methods used include Bag of Words (BoW); which uses the frequency of words to transform text into numerical values, Word2Vec; which generates dense vector representations that capture semantic relationships between words, Term Frequency – Inverse Document Frequency (TF-IDF) which is used to determine the relative value of words in a dataset based on how often they appear, and Principal Component Analysis (PCA) which reduces the number of dimensions while keeping the most useful features.

These ways for extracting features are very important for showing the data correctly, which makes it useful for CNN-based classification models and improving the overall detection accuracy of malware attacks in network traffic.

#### Bag of Words (BoW)

This study utilized CountVectorizer from scikit-learn to implement a BoW model. The CountVectorizer was fitted on the training subset only and then applied to transform validation and test data using the learned vocabulary. The model takes network traffic data as input and uses sparse matrices to represent it, with rows representing records of data and columns that show how often a certain word appears in those records. The model disregards word structure and order but preserves word multiplicity. This is an easy way to do it; all you have to do is take a collection of texts and count how many times each word appears in them. This produces the space of vectors with more than 100 dimensions. This space improves textual data processing by placing vectors representing category variables next to each other in cases when they have comparable contexts^49^.

Mathematically, let V = {V_1_, V_2_, V_3_… V_n_} represent the vocabulary extracted from the dataset. For any given document d, the BoW vector is defined as^50^

3 $$D^{ \to } = {\text{ }}\left[ {Freq{\text{ }}(V_{1} ,{\text{ }}d),Freq{\text{ }}(V_{2} ,{\text{ }}d),Freq{\text{ }}(V_{3} ,{\text{ }}d),{\text{ }} \ldots ,Freq{\text{ }}(V_{n} ,{\text{ }}d)} \right]$$

Where *Freq (V*_*i*,_ *d)* denotes the frequency of the word *V*_*i*_ in document d.

This produces a sparse vector representation of the document, with each dimension representing a vocabulary word and the number of times it’s referenced throughout the text. The model may concentrate on the frequency and existence of significant phrases that could point to malicious activity in network traffic thanks to this representation.

#### Term Frequency-Inverse Document Frequency (TF-IDF)

In this study, the TF-IDF technique is utilized as one of the feature extraction methods to convert textual data into numerical representations that highlight the importance of each word within individual network traffic records. TF-IDF relies on two fundamental concepts: One way to find out the number of times a word shows up in a document is by looking at its term frequency (TF).

To find out how infrequent or instructive a term is throughout all of the documents, you can use the Inverse Document Frequency (IDF) metric.

The frequency of a categorical variable in our corpus was determined using TF-IDF, as demonstrated in Eq. (4)^3^, as the score for word i in document j. We fitted the TfidfVectorizer on the training corpus to learn the vocabulary and IDF statistics and then transformed the validation and test subsets using the same fitted vectorizerv

4 $$TFIDF{\text{ }} = {\text{ }}TF_{{(t,d)}} ~ * IDF_{{(t)}}$$

Where IDF is Inverse Document Frequency, TF is Term Frequency, t is a term or a quantitative variable, and d is Document or a corpus or a subset that contains only categorical variables.

5 $$TF~\left( {t,d} \right)~~~ = \frac{{Term~i~frequency~in~document}}{{Total~words~in~document}}$$

6 $$IDF~\left( t \right) = ~\log _{2} \left( {\frac{{Total~documents}}{{document~with~term~t~}}} \right)$$

The above Eqs. (5–6) can be generalized into one component. The actual mathematical formula becomes as shown in Eq. (7)

7 $$W_{{(t,d)}} = {\text{ }}tf_{{(t,d)}} *\log \frac{N}{{df\left( t \right)~}}$$

To clarify, tf (t, d) is the count of times t appears in document d, df (t) is the count of documents that contain t, and N is the total count of documents.

This technique reduces the impact of frequent and repeating words that might not have any real meaning while allowing the model to give greater weights to significant and distinctive terms found in network traffic logs. As a result, TF-IDF enhances classification models’ ability to identify harmful activities and cyberattacks^51^.

#### Word2Vec

The Word2Vec method was utilized in this study for converting fractional textual data into numerical representations because it is able to vectorize words and determine their relative importance and enables the analysis of word usage patterns in texts to show contextual relationships between words^52,53^. As a result, text data representation for deep learning models is of higher quality. This was accomplished by segmenting the texts into words using Generate Similar (Gensim) tools that ensured a consistent and uniform segmentation. Gensim is a Python library for semantic tools that is open source^54^. Then the Word2VEC model was trained on the textual feature corpus using a vector size of 100, a context window of 5, and a minimum word occurrence threshold of 1 to ensure comprehensive learning of network traffic semantics.

Using the target word as input, Word2Vec’s Skip-Gram model attempts to optimize the average log probability of predicting the context words^55^, as illustrated in Eq. (8) :

8 $$\frac{1}{T}~~~\sum\nolimits_{{t = 1}}^{T} {} \sum\nolimits_{{ - c \le j \le c,~j \ne 0}}^{{}} {} \log P\left( {Wt + j|Wt} \right)~~$$

Where: The total count of words in the corpus is represented by T, c Denotes the size of the context window and the conditional probability of a context word depending on the target word is denoted as.

p(wt + j│wt). Equation (9) shows the process for calculating this conditional probability using the softmax function:

9 $$p\left( {w_{o} |w_{1} } \right) = \frac{{\exp \left( {\dot{v}_{{w_{{o~~}} }} ~v_{{w1~}} } \right)}}{{\mathop \sum \nolimits_{{w = 1}}^{w} \exp \left( {\dot{v}_{{w_{{o~~}} }} ~v_{{w1~}} } \right)}}$$

By ensuring that words with comparable contexts are mapped to similar vector representations, this mathematical formula improves the model’s comprehension of the meaning contained in textual features by capturing the syntactic and semantic links between words.

####  Principal component analysis (PCA)

This research used PCA to extract features from the dataset in an effort to lower their dimensionality without losing any of the important information. By transforming the initial features into a new collection of vertical components, or principal components, that get the most out of the data, PCA reduces computing complexity, eliminates noise, and lowers the danger of overfitting^56^. PCA is implemented to find a smaller set of features that, with the least amount of information loss, show the primary data in a subspace with fewer dimensions^57^. In this paper each text instance was transformed into a 100-dimensional numerical vector, with missing words replaced by zero vectors to maintain data consistency. After that, PCA was fitted on the training subset only to determine the principal components capturing the highest variance. The learned projection matrix was subsequently applied to validation and test subsets. Selecting this number of features was based on dimensionality reduction analysis to ensure that the most important information that reflects the variance within the data is retained while reducing computational complexity and aims to reach the perfect equilibrium between preserving essential information and avoiding the overfitting problem^56^.

### Feature selection

Importantly, following preprocessing is feature selection, a method for removing characteristics from the dataset that do not contribute to or solve the problem at hand^45^. The characteristics are chosen using approaches from Recursive Feature Elimination and correlation analysis.

#### Recursive feature elimination (RFE)

RFE is a feature selection algorithm that works by gradually removing unimportant features^58^. This research makes use of RFE with a Random Forest classifier. This technique removes the elements that are least significant in an iterative manner and retains the top most significant attributes that contribute to network attack detection^59^. After multiple iterations, the 20 most significant features are selected, ensuring that only the most relevant attributes for network attack detection are retained. After that, the deep learning models are trained using these attributes as input as well. RFE was performed exclusively on the training subset within each experiment to identify the most relevant features. The selected feature subset was then applied consistently to validation and test data.

####  Correlation analysis

**Fig. 2:**
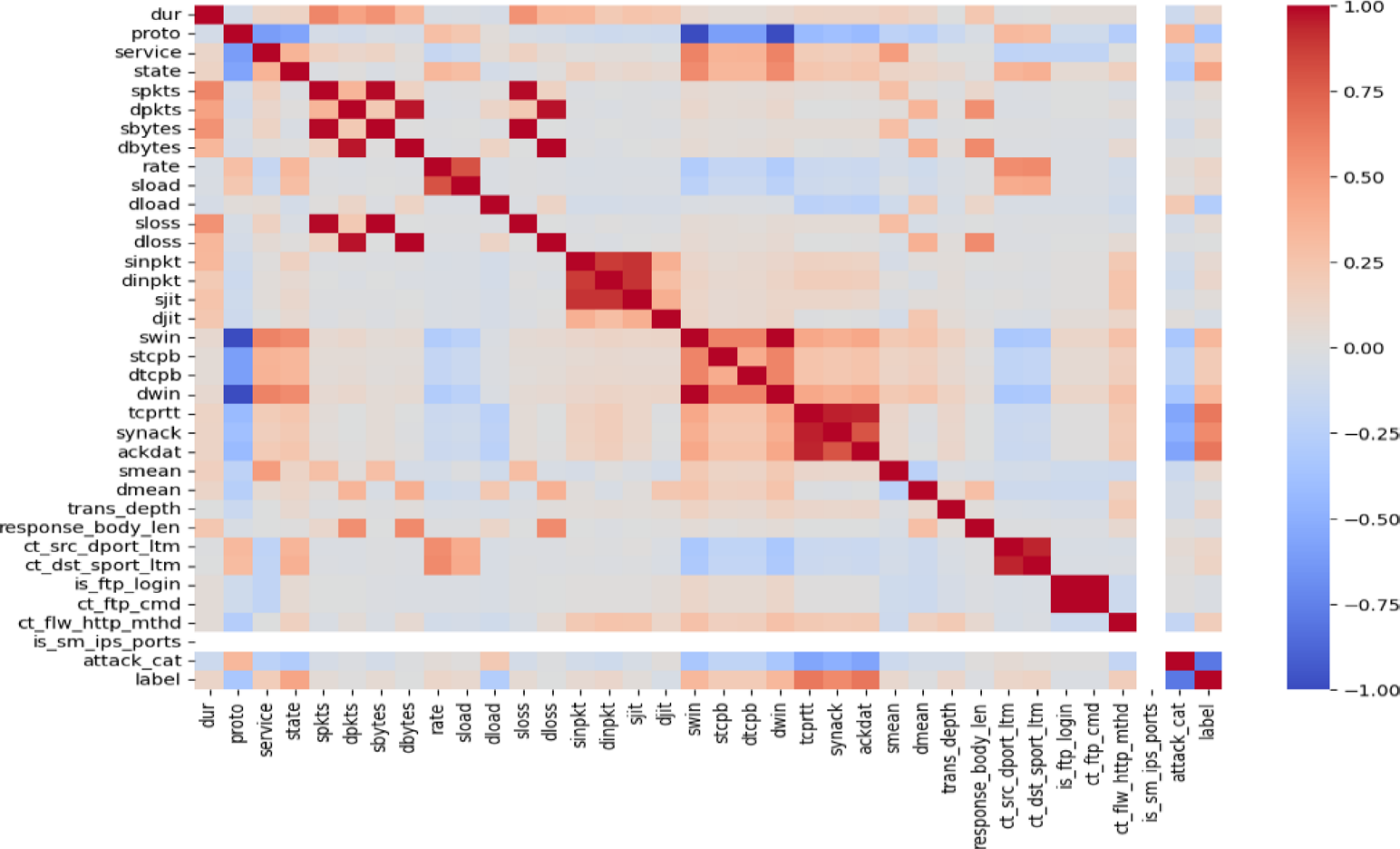
Correlation analysis of the dataset.

Correlation analysis is carried out utilizing heat map visualization to analyze the relationships between different features. Finding any problems with multicollinearity is made easier by the correlation matrix’s insights into the relationships of the characteristics. Features with very high correlations may lead to redundancy, which could affect the capacity of the model for effective generalization^45^. Understanding these relationships helps us keep the dataset balanced and devoid of extraneous characteristics that can impair classification accuracy. A heatmap of correlation analysis of the data is shown in Fig. 2.

All feature extraction, feature selection, normalization, and dimensionality reduction procedures were fitted exclusively on the training data within each split. The learned transformations were then applied to the corresponding validation and test sets to prevent data leakage and ensure fair evaluation.

### Convolutional neural network (CNN) architectures

Due to the fact that CNNs are able to automatically extract hierarchical features from raw input, a number of applications that utilize deep learning make considerable use of them. Within the scope of this section, the CNN-based models for the detection of malware traffic are covered. Table 2 presents a systematic comparison of the four M-Block architectures employed in each model. A thorough explanation of the results of the experiment and evaluation measures is given in the section that follows.

#### M1-block

**Fig. 3:**
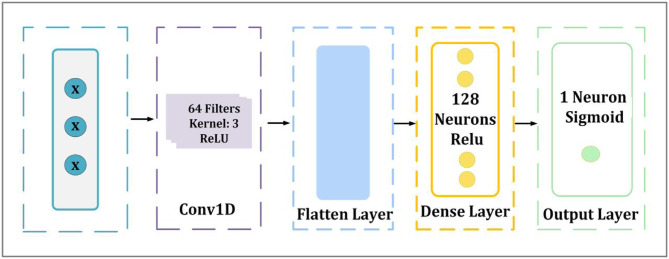
Architecture of the proposed M1-block.

The first suggested model is made as a deep learning architecture for detecting through binary classification of network activities, which consists of four layers and incorporates convolutional feature extraction, flatten, two dense layers and the detailed architecture is presented in Fig. 3. Conv1D, which stands for “one-dimensional convolutional layer,” is the first layer of the model. It employs the Rectified Linear Unit (ReLU) activation function, and it possesses 64 filters and a kernel size of 3. Deep neural networks are able to acquire sophisticated patterns and representations as a result of the ReLU activation function, which introduces non-linearity into the learning process. Deep neural networks’ optimization and performance enhancement depend on this non-linearity^60^. In the context of deep learning, the Eq. (10) illustrates how ReLU functions can be utilized^61^

10 $$\frac{1}{T}~~~\sum\nolimits_{{t = 1}}^{T} {} \sum\nolimits_{{ - c \le j \le c,~j \ne 0}}^{{}} {} \log P\left( {Wt + j|Wt} \right)~~$$

This layer is in charge of identifying significant patterns and capturing spatial dependencies in the incoming data. This is followed by the flatten layer which transforms the output into a one-dimensional vector in order to get the retrieved features ready for dense processing. To train the model to understand complicated feature representations, the first dense fully connected layer has 128 neurons activated by ReLU.

Lastly, the output layer was made up of a single neuron that had a sigmoid activation function. This configuration allowed for binary classification, which was necessary for the detection of malicious software. When it comes to neural network binary classification tasks, the sigmoid activation function is often utilized because it has the capability to turn input values into a probability-like output between 0 and 1, which assists with decision-making^61^. This ability is important since it helps with decision-making. Sigmoid functions can be applied in the context of deep learning, as demonstrated by the Eq. (11), which shows this utilization.

11 $$\partial \left( x \right){\text{ }} = \frac{1}{{1 + ~e^{{ - x}} }}$$

####  M2-block

**Fig. 4:**
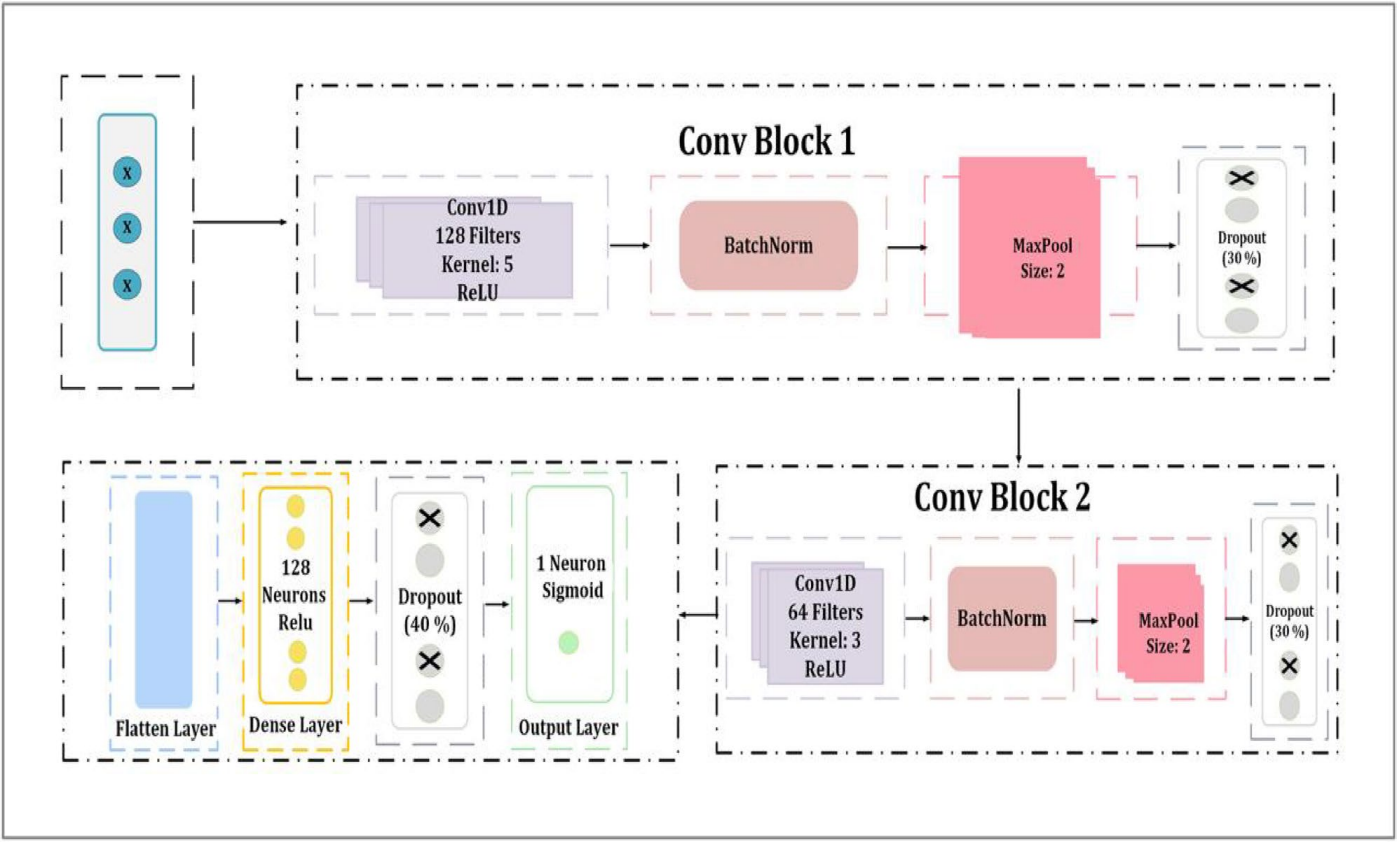
Architecture of the proposed M2-block.

The second suggested architecture introduces additional enhancements, including BatchNormalization, MaxPooling1D, and Dropout layers. It is designed to enhance feature extraction while incorporating regularization algorithms to enhance generalization and avoid overfitting. Figure 4 shows the architecture of the second CNN model, which introduces additional convolutional blocks. At the beginning of the model is a Conv1D. This layer employs the ReLU activation function and possesses 128 filters, with a kernel size of 5. Immediately after this, a batch normalization layer is applied, which is responsible for normalizing activations in order to stable training and accelerate convergence. Batch Normalization, often known as BatchNorm, is a technique that improves the training effectiveness of deep neural networks, in particular CNNs. It has been shown empirically to improve accuracy, stability, and performance; nevertheless, it is unknown why these gains occur^62^. Every feature is processed individually per mini-batch using BatchNorm. Equations (12) and (13) show how we determine the mean and variance for each characteristic xj.

12 $$\:{\upmu\:}\mathrm{j}\:=\:\frac{1}{m}\:\sum\:_{i=1}^{m}xij$$

13 $$\partial _{j} 2 = 1/m\sum\nolimits_{{(i = 1)}}^{m} {\left( {xij - {\text{ }}\mu j} \right)} ^{2}$$

In that order. The z-score is derived from these values.

14 $$X_{i} = \frac{{xij - ~\mu j}}{{\sqrt {\partial j2 + \varepsilon } ~~~~~~}}$$

In this case, ε is a tiny constant that helps to keep the output steady. At last, we determine the result.

15 $$\:yi\:\left(\mathrm{m}\right)\:={\gamma\:}_{j}\:\:\frac{{x}_{j\:\left(m\right)-\:{\mu\:}_{j\:}^{t}}}{{\alpha\:}_{j}^{t}}\:\:+\:{\beta\:}_{j}$$

Where $\:{\mu\:}_{j\:}^{t}$ and $\:{\alpha\:}_{j}^{t}$ are, respectively, the mean and variance of the outputs of the neuron in the target domain.

A MaxPooling1D layer is then used to reduce dimensionality while preserving essential information. The max pooling approach is a straightforward and popular CNN technique that finds the element that is the most abundant in each pooling zone to optimize the spatial size of feature maps and provide translation invariance^63^. Equation (16) shows how max pooling can be used in CNN. Since the feature map’s values reflect features, it is imperative that significant large values are preserved in the event that the size of the feature map is reduced. To prevent the loss of maximum values during spatial sampling, the concept of max pooling ensures that the highest possible values within the window are reproduced and maintained^64^.

16 $$F_{{\max }} \left( X \right)_{{ = ~}} \max _{{i~}} x_{{i~}}$$

Adding a second Conv1D layer with 64 filters and a kernel size of three, followed by another batch normalization and maximum data pooling, further enhances the feature extraction process and guarantees the successful learning of hierarchical representations. A 30% dropout following the first convolutional block and a second 30% dropout following the second convolutional block are included at different times to reduce overfitting. This is followed by the flatten layer which transforms the output into a one-dimensional vector in order to get the retrieved features ready for dense processing. To train the model to understand complicated feature representations, the first dense fully connected layer has 128 neurons activated by ReLU. A 40% dropout layer is applied before the final layer. A single neuron with a sigmoid activation function made up the output layer, which was the final layer. By combining them, binary classification was made possible, which is essential for malware detection.

#### M3-block

**Fig. 5:**
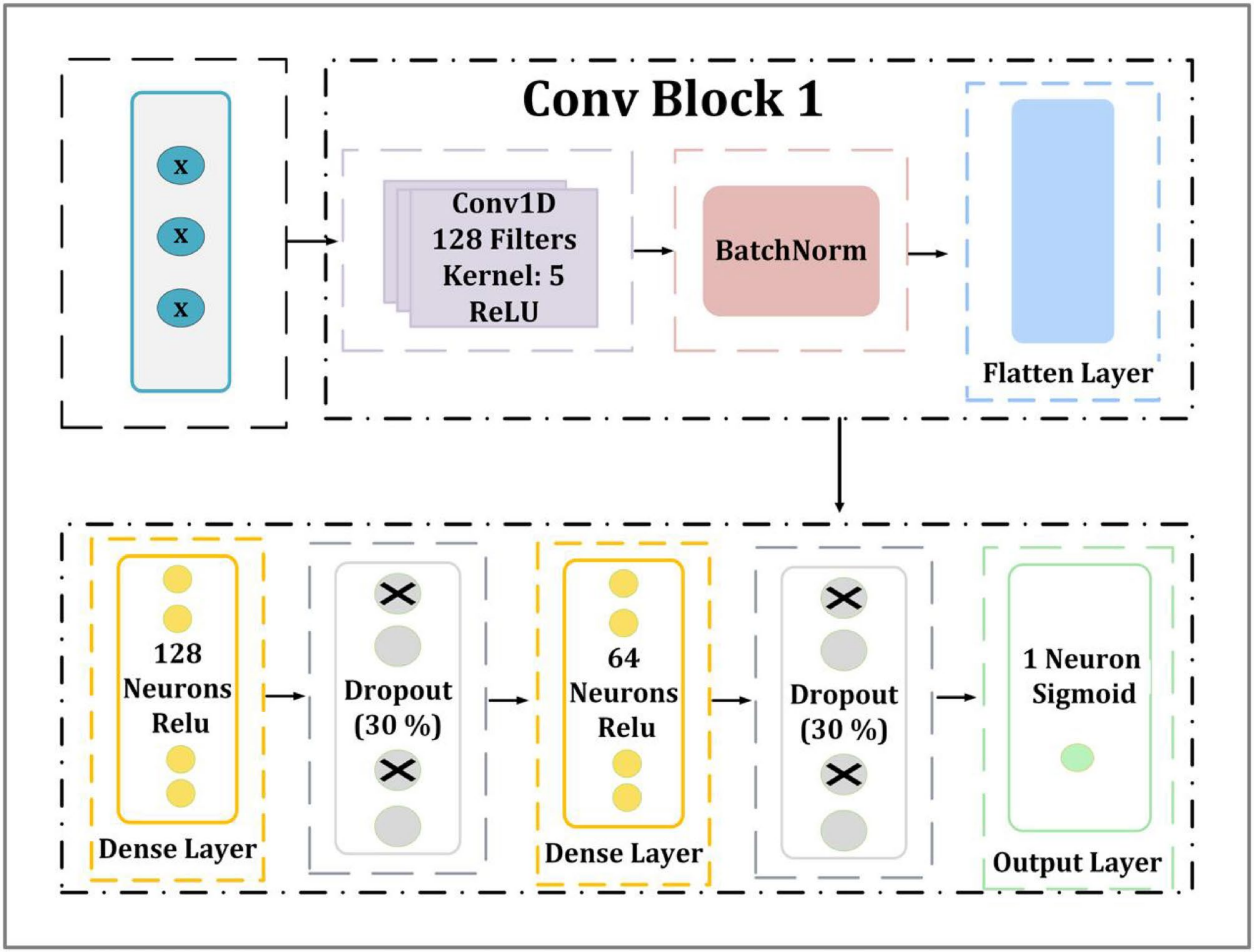
Architecture of the proposed M3-block.

The third architecture introduces additional enhancements. It is made as a deep learning architecture for detecting through binary classification of network activities, which consists of eight layers as shown in Fig. 5 and incorporates convolutional feature extraction, normalization, dense layers, and regularization approaches to improve performance and avoid overfitting. At the beginning of the model is a Conv1D. This layer employs the ReLU activation function and possesses 64 filters, with a kernel size of 3. Immediately after this, a batch normalization layer is applied, which is responsible for normalizing activations to stable training and accelerate convergence. The flatten layer transforms the output into a one-dimensional vector in order to get the retrieved features ready for dense processing. To train the model to understand complicated feature representations, the first dense fully connected layer has 128 neurons activated by ReLU. After that, a dropout layer was combined at a 30% rate, and the model’s adaptation is improved by adding a dense layer with 64 neurons and a dropout rate of 0.3. In our model design, 128 neurons were selected in the first hidden layer and 64 in the second to strike a compromise between how complicated the model is and how well it can generalize. This configuration proved to be efficient during the model selection process, providing sufficient learning ability from the data without causing overfitting. Lastly, the output layer had one neuron that used a sigmoid activation function.

####  M4-block

**Fig. 6:**
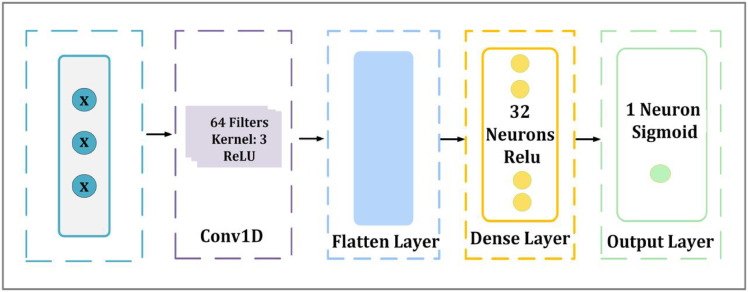
Architecture of the proposed M4-block.

Building upon the previous M1-Block, a number of changes were made to the model in order to improve feature extraction and classification capabilities. Unlike M1-Block, which received the input shape directly, this architecture restructures the dataset into (samples, 50, 1) in order to better match the data with the 1D CNN frameworks and ensure efficient spatial feature extraction. By reducing the fully connected layer size from 128 neurons to 32 neurons, the network’s learning ability was maintained while its complexity was optimized. The Conv1D layer structure remained similar, maintaining 64 filters with a kernel size of 3, ensuring robust feature extraction. Figure 6 illustrates the architecture of M4-Block and its changes.

The strength of the proposed M-blocks lies in their carefully designed, task-specific architecture, which directly contributed to the improved detection results. Unlike standard CNN architectures that are often adopted in a generic manner for malware detection, the proposed M-blocks are explicitly tailored for IoT network traffic analysis.

Specifically, the use of Conv1D layers enables effective capture of local sequential and statistical traffic patterns, which are critical characteristics of network-based malware behavior. Each M-block integrates convolutional feature extraction, batch normalization for training stability, dense layers for learning high-level feature interactions, and dropout-based regularization to mitigate overfitting in IoT datasets.

Moreover, multiple M-block configurations with varying depths and neuron sizes were systematically designed and experimentally evaluated. The selected M-blocks demonstrated superior performance and generalization compared to other tested architectures, confirming that the proposed block design plays a key role in achieving the reported results.

###  Dropout configuration

Dropout layers with rates of 0.3 were incorporated within the convolutional and dense blocks of the proposed architectures to mitigate overfitting while preserving sufficient feature learning capacity. This value was selected based on empirical observations during the model selection process and was found to provide stable convergence and improved generalization across different M-block designs. Higher dropout rates adversely affected feature representation, whereas lower rates resulted in reduced regularization. The adopted configuration represents a practical balance for compact CNN models operating on network traffic data.

### Proposed models

To investigate the influence of architectural complexity and optimization strategies on the classification performance, five distinct model variants were developed. These models are designed with different processing mechanisms and block configurations .These models are named DLRCM1-MODEL, DLRCM2-MODEL, DLRCM3-MODEL, BTWMM3-MODEL, and MTWPM4-MODEL. Each model introduces specific architectural adjustments intended to evaluate how different structural choices contribute to performance improvements. A detailed description of each architecture is provided in the following subsections.

In the DLRCM1-MODEL model, we began by cleaning the data by removing duplicate records, addressing missing values, and correcting inconsistent entries. This step helped ensure data quality and noise-free performance, enhancing the reliability of training and evaluation. Next, categorical features were converted to numerical values ​​using label encoding, which helped preserve the implicit relationships between text values ​​and ensure their compatibility with deep learning models. We utilized RFE to determine the relative importance of each feature and how it affected the classification process so we could prioritize them. By eliminating features with strong correlations, correlation analysis improved model performance by cutting down on redundant data. Finally, the features were then used to train on M1-Block architecture.

In the DLRCM2-MODEL model, we began by cleaning the data by removing duplicate records, addressing missing values, and correcting inconsistent entries. This step helped ensure data quality and noise-free performance, enhancing the reliability of training and evaluation. Next, categorical features were converted to numerical values ​​using label encoding, which helped preserve the implicit relationships between text values ​​and ensure their compatibility with deep learning models. We utilized RFE to determine the relative importance of each feature and how it affected the classification process, so we could prioritize them. By eliminating features with strong correlations, correlation analysis improved model performance by cutting down on redundant data. Finally, the features were then used to train on M2-Block architecture.

In the DLRCM3-MODEL model, we began by cleaning the data by removing duplicate records, addressing missing values, and correcting inconsistent entries. This step helped ensure data quality and noise-free performance, enhancing the reliability of training and evaluation. Next, categorical features were converted to numerical values ​​using label encoding, which helped preserve the implicit relationships between text values ​​and ensure their compatibility with deep learning models. We utilized RFE to determine the relative importance of each feature and how it affected the classification process, so we could prioritize them. By eliminating features with strong correlations, correlation analysis improved model performance by cutting down on redundant data. Finally, the features were then used to train on M3-Block architecture.

In the BTWMM3-MODEL model, three feature extraction techniques, BoW, TF-IDF, and Word2Vec were combined for enhancing the representation of text network traffic data features. Each technique added a distinct feature to the overall feature space. BoW offered a straightforward representation based on word frequency, but TF-IDF enhanced it by giving each word an important weight determined by how unique it was in the dataset. Word2Vec captured the semantic relationships between words by representing them in a dense vector space. After combining the features from all three approaches into a single feature matrix, MinMax scaling was used to standardize the range of values and avoid scaling anomalies in order to guarantee a thorough text representation. Finally, the features were subsequently fed into the M3-Block architecture for training.

In the MTWPM4-MODEL model, initially, MinMaxScaler was used to normalize all numerical features, ensuring constant convergence throughout training by scaling them between 0 and 1. We used Word2Vec and TF-IDF for textual data. After preprocessing we used PCA so, dimensionality was decreased, which kept the most significant characteristics while removing extraneous information and increasing model generalization and feature representation. Finally, the features were subsequently fed into the M4-Block architecture for training.

### Training options

All proposed models in this study were trained using the Adam optimizer with a fixed learning rate of 0.001, as it provides a good balance between convergence speed and stability. According to a study in^65^, 0.001 is a commonly used default value in deep learning applications because it guarantees steady updates without excessive oscillations. In all models, we used binary cross-entropy as the objective function to optimize classification performance. The difference between the predicted labels and the actual labels is computed by this function, making it ideal for probabilistic models^66^. The definition of the binary cross-entropy loss (BCELoss) function is as shown in Eq. (17)^67^

17 $$~BCLoss = \frac{1}{T}\mathop \sum \limits_{{i = 1}}^{T} [(x_{i} ~\log \left( {p\left( {x_{i} } \right)} \right) + \left( {1 - x_{i} } \right)~\log \left( {1 - p\left( {x_{i} } \right)} \right)]$$

Where T is the sample size and x_i is the true class label (a value between 0 and 1), The projected probability is denoted as p(x_i), and the natural logarithm is represented by log.

Training was conducted for a limited number of epochs to ensure sufficient learning while avoiding overfitting. The model may underfit if there are too few epochs, failing to acquire intricate patterns, or overfit if there are too many, memorizing training data but not generalizing well^68^. A split of 80% training and 20% validation was consistently used across all models. Batch sizes varied (32 or 64) depending on the design to balance computational efficiency and generalization.

To further improve convergence, some models applied a ReduceLROnPlateau callback, which dynamically slows down the rate of learning when the validation loss reaches its peak .According to the study in^69^, This technique ensures a suitable learning rate for the optimization process by tracking the verification accuracy and reducing the learning rate when the accuracy improvement interrupts. In particular, to promote better weight updates and keep the model from becoming trapped in local minima, the rate of learning was decreased 50% if the validation loss stayed constant for three consecutive epochs. To prevent excessive reductions and guarantee that training proceeds well, a minimum threshold of 1e-6 was established. This method improves generalization performance, reduces overfitting, and stabilizes training. A summary of the exact training parameters used in each CNN model is provided in Table 2.

**Table 2:** Comparative analysis of CNN architectures Training settings for the five CNN models.

Features	M1-Block	M2-Block	M3-Block	M4-Block
Input Shape	(20,1)	(20,1)	(20,1)	(50, 1)
Conv1D	1 (64 filters)	2 (128→64 filters)	1 (64 filters)	1 (64 filters)
BatchNorm	--	2	1	--
MaxPooling1D	--	(2, size = 2)	--	--
Dense Layers	1 Hidden(128)	1 Hidden(128)	2 Hidden (128→64)	1 Hidden(128)
Dropout	--	3	2	--
Loss Function	BCE	BCE	BCE	BCE
Optimizer	Adam	Adam	Adam	Adam
Learning Rate	0.001	0.001	0.001	0.001
Epochs	20	30	30	20
Batch Size	32	64	32	32
Callback (LR scheduling)	--	--	ReduceLROnPlateau	--

## Environment

To ensure accurate evaluation of our suggested models, we organized the experimental environment for testing by picking a suitable dataset, establishing data splitting strategies, and employing standard performance metrics. Model performance was enhanced by using the feature selection and dataset preprocessing strategies previously covered. The following section summarizes the dataset used, the data partitioning methodology, and the evaluation metrics adopted in this study.

### Dataset

The UNSW-NB15 dataset was developed by the Australian Centre for Cyber Security (ACCS) in Canberra for the purpose of evaluating intrusion detection systems (IDS). In addition to regular traffic, this dataset contains nine distinct kinds of cyberattacks^70^. The IXIA PerfectStorm tool, which simulated both benign and malicious network traffic, was used to create it. The Cyber Range Lab in Canberra was responsible for recording and converting a hundred gigabytes of raw traffic into four million network flows^71^.

There are a total of nine distinct kinds of attacks in the dataset, each standing for a certain kind of cybercrime. Analysis techniques, such as port scanning, are used to gather network data, and fuzzers assaults aim to crash apps by introducing unexpected inputs. Backdoor attacks include malicious software that grants unauthorized users access to a system. DoS (Denial of Service) attacks overload network capacity to interfere with regular operations. Exploits target system vulnerabilities to gain unauthorized access. Generic attacks seek to use encryption techniques to get around security measures. Reconnaissance involves scanning and probing to collect network details. Shellcode attacks execute malicious code directly in system memory. Lastly, worms are malicious software that can replicate itself and infect multiple systems without the user’s awareness or permission^70^.

The UNSW-NB15 dataset is originally designed for network intrusion detection and provides labeled network flow records belonging to one normal class and multiple attack categories. To reflect practical intrusion detection scenarios, the dataset was formulated as a binary classification problem by mapping all attack categories to a single malicious class, while normal traffic samples were assigned to the benign class, as shown in Table 3. Hence, “malware detection” in this paper refers to detecting malicious network activities manifested in traffic flows rather than identifying specific malware families. Although UNSW-NB15 is not IoT-exclusive, its realistic traffic generation and diverse attack behaviors make it a commonly used benchmark to evaluate traffic-based detection models for IoT-like network scenarios.

**Table 3:** Mapping of UNSW-NB15 labels to binary classes.

UNSW-NB15 Label	Our Label
Normal	Benign
Analysis	Malicious
Fuzzers	Malicious
Backdoor	Malicious
DoS	Malicious
Exploits	Malicious
Generic	Malicious
Reconnaissance	Malicious
Shellcode	Malicious
Worms	Malicious

The dataset was used to identify malware attacks. The dataset was divided into training and testing subsets, stored in Parquet format for efficient data handling. Both subsets were loaded using Pandas, ensuring compatibility with subsequent preprocessing and feature extraction steps.

In this study, the official predefined dataset partitions were strictly preserved. The dataset consists of 175,341 samples in the training set and 82,332 samples in the independent test set. This separation ensures that the final evaluation is conducted on unseen data, improving the reliability of the reported performance. The dataset was processed following a structured split-first strategy. After initial data cleaning, the official training set was further divided using stratified sampling into 80% training and 20% validation. An independent test set was preserved and remained completely unseen during model development. The training subset was used for fitting preprocessing transformations and model learning, while the validation subset was used for hyperparameter tuning and early stopping. The independent test set remained completely unseen during model development and was used only for final performance evaluation.

All preprocessing and feature engineering steps including Bow, TFIDF, Word2vec, MinMaxScaler, and feature selection, were fitted exclusively on the final training subset. The learned transformations were then applied to the validation and test subsets to ensure fair evaluation and prevent data leakage. This split was performed exclusively within the official training file using the train_test_split function from Scikit-learn. Once the Splitting was completed, the network traffic samples were labeled as either malicious or benign. Subsequently, a CNN-based deep learning model was constructed for classification purposes once the data preparation was finalized.

The use of UNSW-NB15 provides a representative benchmark for evaluating traffic-based intrusion detection models, as it captures a wide range of modern attack behaviors under realistic traffic conditions. While the dataset does not fully reflect the heterogeneity and dynamics of real-world IoT deployments, it offers a reliable and widely accepted testbed for assessing malware and intrusion detection performance in IoT-like network scenarios.

**Table 4:** Dataset Partitioning for 5-Fold Cross Validation.

Dataset Samples						
Flod-1	Flod-2	Flod-3	Flod-4	Flod-5	Test	Total
35,068	35,068	35,068	35,068	35,069	82,332	257,673

**Table 5:**
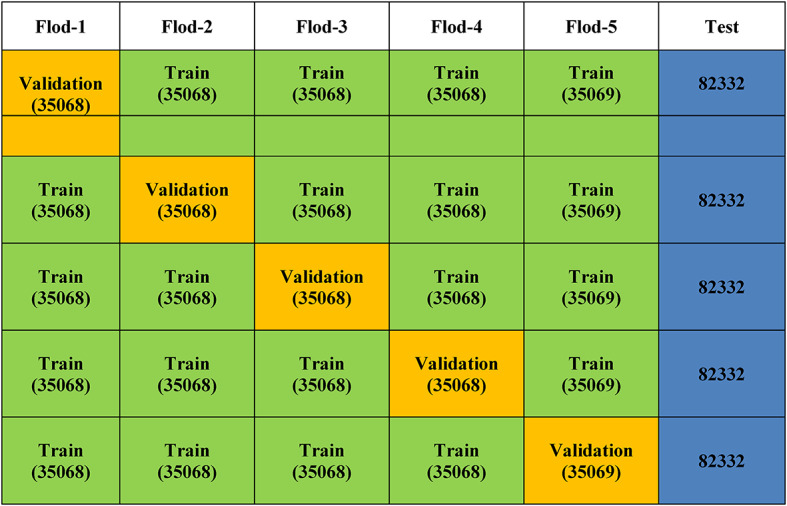
Cross-Validation Pipeline.

Stratified k-fold cross-validation was performed on the training dataset as shown in Table 5 to further evaluate the robustness and generalization capacity of the proposed models. Cross-validation is a better way to estimate how well a model works since it tests it on several data partitions while keeping the original class distribution. This is different from a single train-test split. In this study, stratified 5-fold cross-validation (k = 5) was employed to ensure that both benign and malicious samples were proportionally represented in each fold. Five subsets that didn’t overlap were made from the training dataset. For each iteration, four folds were used to train the model, and the last fold was used to validate it. Five times, this process was performed, with each fold being used once as the validation set. The models were trained separately for each fold, and then the validation fold was used to calculate performance metrics like accuracy, precision, recall, and F1-score.

###  Evaluation metrics

The proposed models were evaluated using key performance indicators, which include recall, accuracy, precision, F1-score, AUC, ROC curves, and a lot of the measures. All of these assessment metrics are obtained from the four parameters that can be found in the confusion matrix, as shown in Table 6, which relies on the computed predicted class in comparison to the actual class.

**Table 6:** Confusion matrix.

		Predicted Class	
		Malware	Benign
Actual Class	Malware	True Positive (TP)	False Negative (FN)
	Benign	False Positive (FP)	True Negative (TN)

Whereas, in the case of TP, all of the data points, both actual and predicted, are true ; FP stands for “False Positives”, which arise when the predicted values are positive even though the original values are false, TN stands for “True Negatives”, which means that both the actual and predicted data values are incorrect, and false negatives, or FN, occur when observed data points are accurate but anticipated data points are incorrect^72^.

The performance of the five CNN models was assessed using multiple evaluation metrics derived from the confusion matrices, including Accuracy (ACC), Precision (PPV), Recall (Sensitivity/TPR), F1 Score, Specificity (TNR), Negative Predictive Value (NPV), and Area Under the Curve (AUC). The detailed values are presented in Table 5.

Accuracy is measured as the sum of two accurate predictions (TP + TN) divided by the total number of data sets (P + N).

18 $$~ACC~ = \frac{{TP~ + ~TN}}{{P + N}}$$

Precision ($\:\mathrm{P}\mathrm{R}\mathrm{E}\mathrm{C}$) or Positive Predictive Value (PPV) is measured as the number of correct positive predictions (TP), divided by the total number of positive predictions (TP + FP).

19 $$PREC~ = \frac{{TP}}{{TP + FP}}$$

Recall or Sensitivity or True Positive Rate (TPR) is defined as the percentage of correct positive predictions (TP) relative to the total number of positive predictions (P).

20 $$REC~ = \frac{{TP}}{P}$$

The F-score is a reliable indicator of the test’s accuracy. It is decided by considering recall and precision simultaneously^73^.

21 $$F - score = 2*\frac{{\Pr ecision*\mathrm{Re} call~}}{{\Pr ecision + \mathrm{Re} call}}$$

Negative Predictive Value (NPV) is the probability that a sample is truly benign (non-attack) given that the model has classified it as benign^74^.

22 $$NPV = \frac{{TN}}{{TN + FN}}$$

Specificity or True Negative Rate (TNR) represents the proportion of benign (non-attack) samples that are correctly classified as benign out of all actual benign samples^74^.

23 $$TNR = \frac{{TN}}{{TN + FP}}$$

Area under the Curve (AUC) is a popular performance metric for problems with binary classification. The chance that a classifier will assign a higher score to a positive observation than a negative one is shown by this metric. A receiver operating characteristic curve (ROC) plots metrics like true positive and false positive values to show how well a classifier performs at different threshold levels. The AUC-ROC measures the area under the ROC curve in two dimensions, as determined by^72^ using the following formula as shown in Eq. (24).

24 $$AUROC = \mathop \smallint \limits_{0}^{1} \frac{{TP}}{P}~~~~d~\left( {\frac{{FP}}{N}} \right)$$

## Results

**Fig. 7:**
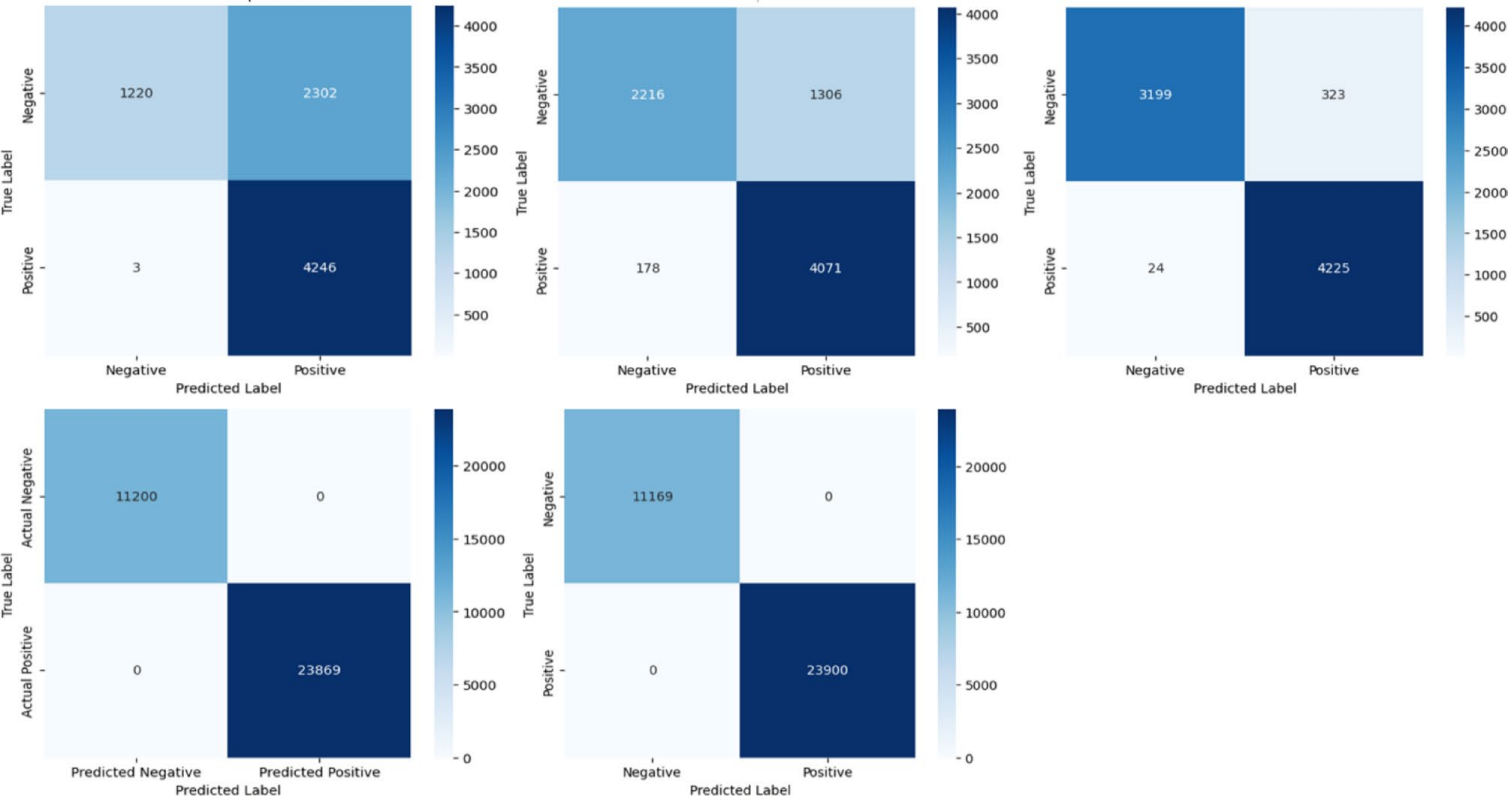
Confusion Matrices of the Proposed Models Evaluated on the UNSW-NB15 dataset: First row from left to right: DLRCM1-MODEL, DLRCM2-MODEL, DLRCM3-MODEL; and the second row: the left is BTWMM3-MODEL and the right is MTWPM4-MODEL.

In this part, we compare and contrast several preprocessing strategies, feature extraction and selection approaches, and convolutional neural network (CNN) architectures. The models were evaluated using accuracy, area under curve precision, f1-score, precision, and recall. The effectiveness of classification was examined using a confusion matrix, which demonstrated the model’s ability to discern between safe and dangerous network traffic. Our goal is to compare various models’ efficacy. There were five CNN models tested on the UNSW-NB15 dataset, and their confusion matrices for validation can be seen in Fig. 7. Each grid shows how the classifications are spread out across TP, TN, FP, and FN. While this image shows accurate numbers, the matrices demonstrate the models’ strengths and shortcomings at various phases of optimization.

DLRCM1-MODEL has excellent detection performance, with a large number of True Positives (4246). However, this comes at the expense of a huge number of False Positives (2302), implying that a significant quantity of benign traffic was wrongly identified as malicious. This implies that the model is extremely sensitive but lacking in precision. DLRCM2-MODEL makes this trade-off better by cutting down on False Positives by a lot, bringing them down to 1306. But this change raised the number of False Negatives to 178, which suggests that more attacks were missed than in DLRCM1-MODEL. It became more accurate, but it lost recall, which shows that the decision boundary changed. The balance is significantly improved in DLRCM3-MODEL. It performs well at reducing both kinds of errors, with only 323 false positives and 24 false negatives. This balanced distribution shows that DLRCM3-MODEL is the most stable of the first three CNNs. This makes sure that both assaults are detected and innocuous traffic is correctly classified. The performance of CNN BTWMM3-MODELand MTWPM4-MODEL is much better. Both models were almost perfect at classifying, with no False Positives or False Negatives. These findings make it clear how model tuning and design refinement affect CNN’s ability to find malware.

The final cross-validation performance of each model was the average of the five folds. This showed how well the model could predict and how stable it was. The detailed per-fold findings for all the CNN architectures that were tested can be found in Table 7. The proposed models show consistent performance across different folds, as you can see. This shows that the models train in a steady way and are less sensitive to certain data partitions, which means they work well with the supplied dataset. It is important to highlight that cross-validation was only done on the training set. The independent test set was kept for the final evaluation. This technique prevents data leaking and guarantees an equitable evaluation of model generalization. The current work does not include cross-dataset or real-world traffic evaluation because of limitations in dataset availability. However, the cross-validation analysis that was done makes the experimental evaluation stronger by showing that it is stable over different training-validation splits.

**Table 7:** Stratified 5-fold cross-validation results.

Method	Metrics	Flod-1	Flod-2	Flod-3	Flod-4	Flod-5	Average
DLRCM1-MODEL	Train-Acc	0.5739	0.6	0.606	0.5996	0.6019	0.596
	Val-Acc	0.5754	0.5963	0.6054	0.6053	0.5989	0.5962
	Val-F1	0.7	0.7095	0.7148	0.7147	0.5529	0.7102
	Val-Precision	0.5388	0.5516	0.5571	0.5571	0.712	0.5515
	Val-Recall	0.9986	0.9941	0.997	0.9967	0.9996	0.9972
DLRCM2-MODEL	Train-Acc	0.7842	0.7972	0.8017	0.7406	0.7817	0.78108
	Val-Acc	0.7848	0.7944	0.8063	0.7426	0.7813	0.7819
	Val-F1	0.8124	0.8141	0.8263	0.7163	0.8069	0.7952
	Val-Precision	0.7155	0.7379	0.7441	0.7901	0.7179	0.7411
	Val-Recall	0.9397	0.9079	0.9289	0.6552	0.9211	0.8706
DLRCM3-MODEL	Train-Acc	0.887	0.8872	0.8009	0.9613	0.8873	0.8847
	Val-Acc	0.8878	0.888	0.8027	0.9597	0.8867	0.885
	Val-F1	0.8975	0.898	0.808	0.9585	0.8966	0.8917
	Val-Precision	0.8203	0.8191	0.781	0.9804	0.819	0.844
	Val-Recall	0.9907	0.9937	0.837	0.9376	0.9905	0.9499
BTWMM3-MODEL	Train-Acc	1.0	1.0	1.0	1.0	1.0	1.0
	Val-Acc	1.0	1.0	1.0	1.0	1.0	1.0
	Val-F1	1.0	1.0	1.0	1.0	1.0	1.0
	Val-Precision	1.0	1.0	1.0	1.0	1.0	1.0
	Val-Recall	1.0	1.0	1.0	1.0	1.0	1.0
MTWPM4-MODEL	Train-Acc	1.0	1.0	1.0	1.0	1.0	1.0
	Val-Acc	1.0	1.0	1.0	1.0	1.0	1.0
	Val-F1	1.0	1.0	1.0	1.0	1.0	1.0
	Val-Precision	1.0	1.0	1.0	1.0	1.0	1.0
	Val-Recall	1.0	1.0	1.0	1.0	1.0	1.0

In conclusion, the five CNN models show how design tuning improves performance: from Model 1, which is too sensitive but not accurate, to Models 2 and 3, which are in a trade-off phase, to Models 4 and 5, which are highly optimized and achieve perfect classification.

**Table 8:** Performance Evaluation Metrics of The Proposed CNN Models.

Model	ACC	Precision (PPV)	Recall (Sensitivity / TPR)	F1 Score	Specificity (TNR)	NPV	AUC
DLRCM1-MODEL	70.34	64.84	99.93	78.65	0.6484	0.9992	0.6941
DLRCM2-MODEL	80.9	75.71	95.81	84.58	0.6291	0.9256	0.9045
DLRCM3-MODEL	95.53	92.9	99.44	96.06	0.908	0.9925	0.9573
BTWMM3-MODEL	100	100	100	100	1	1	1
MTWPM4-MODEL	100	100	100	100	1	1	1

**Fig. 8:**
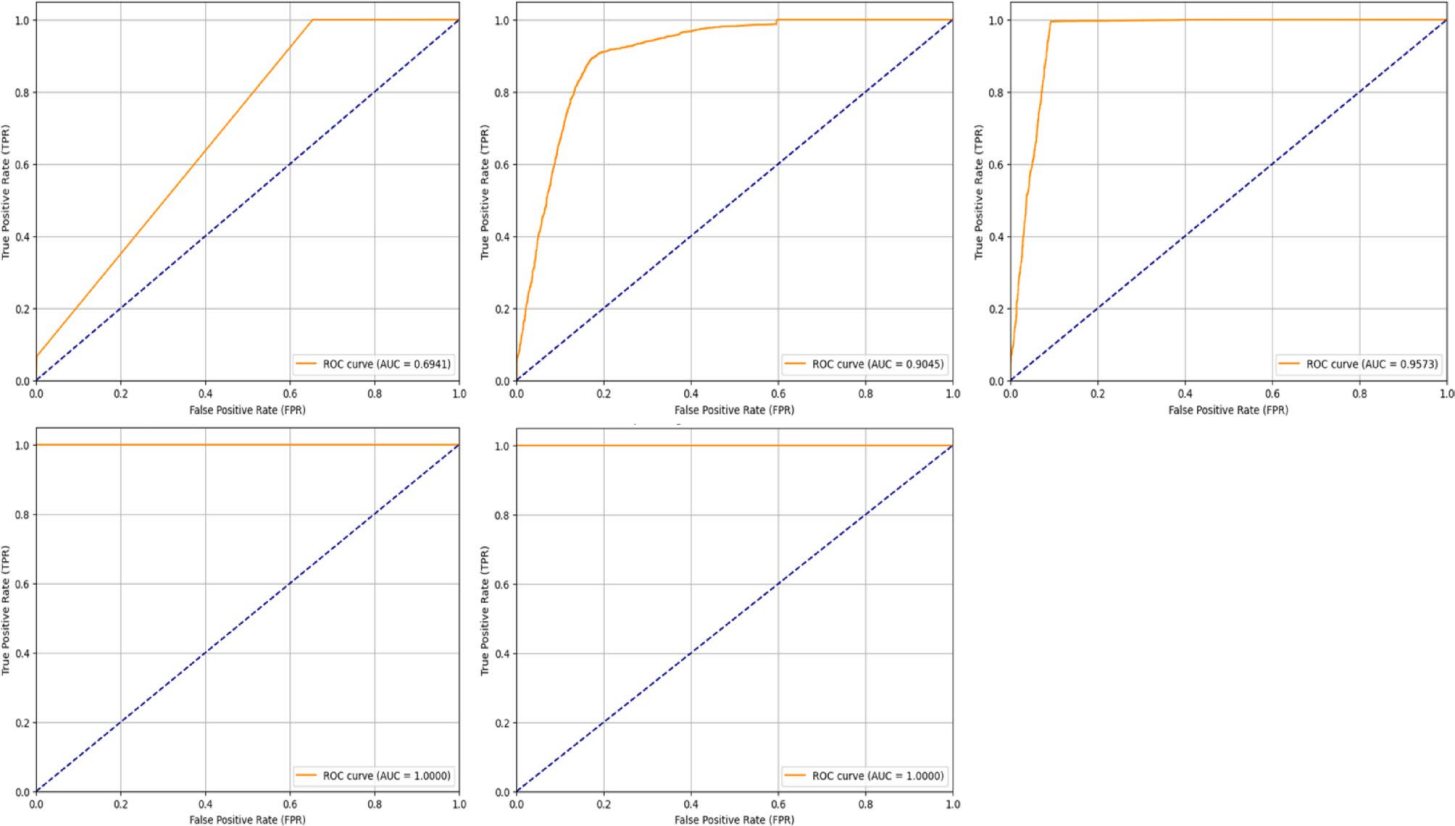
The ROC Curves of The Proposed CNN models: First row from left to right: DLRCM1-MODEL, DLRCM2-MODEL, DLRCM3-MODEL; and the second row: the left is BTWMM3-MODEL and the right is MTWPM4-MODEL.

Table 8 demonstrates that DLRCM1-MODEL achieved the lowest accuracy (70.34%) with a weak AUC value of 0.6941, which reflects inadequate classification abilities. Overall, DLRCM1-MODEL achieved the lowest accuracy. The accuracy was increased to 80.9% using DLRCM2-MODEL, which also displayed a significant increase in AUC (0.9045). With an area under the curve (AUC) of 0.9573, DLRCM3-MODEL was able to achieve a balanced trade-off between precision (92.9% or higher) and recall (99.44%), hence considerably improving the performance. The corresponding ROC curves are shown in Fig. 8. In conclusion, BTWMM3-MODEL and MTWPM4-MODEL got perfect scores across all criteria, showing that they were able to classify every single item perfectly.

The AUC values confirm the numerical results in Table 8. DLRCM1-MODEL achieved the lowest performance (AUC = 0.6941), indicating weak discriminative ability. DLRCM2-MODEL improved substantially with an AUC of 0.9045, while DLRCM3-MODEL demonstrated strong robustness with an AUC of 0.9573. BTWMM3-MODEL and MTWPM4-MODEL both achieved perfect results (AUC = 1.0), reflecting flawless classification without any misclassifications on the test set.

As a conclusion, the evaluation demonstrates that there is a discernible progression in performance across all of the CNN models. Early models, such as DLRCM1-MODEL and DLRCM2-MODEL, had difficulty distinguishing between precision and recall, whereas DLRCM3-MODEL was able to successfully strike a balance between the two. A flawless classification was achieved by the final models (BTWMM3-MODEL and MTWPM4-MODEL) on the test set, indicating that architectural optimization improved the CNN’s capability to identify malware in the UNSW-NB15 dataset. To clearly position the performance of the proposed model within the existing literature, Table 10 provides a concise comparative summary between this study’s results and those reported in previous work. This comparison offers a direct quantitative view that highlights how the proposed model aligns with or surpasses earlier findings. The proposed model demonstrates a strong performance relative to the results presented in previous studies.

**Table 9:** Comparative Performance Analysis of Proposed Models Against Prior Research.

RF	TP	FP	FN	TN	ACC	Precision (PPV)	Recall (Sensitivity / TPR)	F1 Score	Specificity (TNR)	NPV	AUC
^28^	45,949	275	115	36,659	99.53	99.53	99.53	99.53	99.25	99.69	99.985
^29^	85,728	87	208	46,413	99.67	99.62	99.78	99.7	--	--	--
^30^	-	-	-	-	98.18	-	-	-	-	-	-
^31^					98.41	98.41	98.56	--	--	--	--
^32^	-	-	-	-	98.29	97.38	-	-	-	-	-
^33^	-	-	-	-	98	0.983	-	98.97	-	-	0.983
^34^	-	-	-	-	98.36	100	99.96	99.95	-	-	-
^35^	-	-	-	-	99.98	99.98	99.98	99.98	-	-	-
^2^	-	-	-	-	97.67	97.72	97.68	97.7	-	-	-
^36^	-	-	-	-	99.66	-	-	-	-	-	-
^37^	-	-	-	-	97.49	97.7	97.17	97.23	-	-	0.9934
^38^	-	-	-	-	93.3		87.5		-	-	-
^39^	-	-	-	-	94.44	92	100	96	-	-	-
^40^	-	-	-	-	98	-	98.2	98	-	-	-
^41^	-	-	-	-	95.38	96.99	-	94.71	-	-	-
^42^	419	4	1	416	99.4	99.4	99.4	99.4	-	-	0.997
^43^	-	-	-	-	98.29	97.38	-	-	-	-	-
**DLRC** **M1-MODEL**	**4246**	**2302**	**3**	**1220**	**70.34**	**64.84**	**99.93**	**78.65**	**0.6484**	**0.9992**	**0.6941**
**DLRC** **M2-MODEL**	**4071**	**1306**	**178**	**2216**	**80.9**	**75.71**	**95.81**	**84.58**	**0.6291**	**0.9256**	**0.9045**
**DLRC** **M3-MODEL**	**4225**	**323**	**24**	**3199**	**95.53**	**92.9**	**99.44**	**96.06**	**0.908**	**0.9925**	**0.9573**
**BTWM** **M3-MODEL**	**23,869**	**0**	**0**	**11,200**	**100**	**100**	**100**	**100**	**1**	**1**	**1**
**MTWP** **M4-MODEL**	**23,869**	**0**	**0**	**11,200**	**100**	**100**	**100**	**100**	**1**	**1**	**1**

Significance values are in bold.

### Computational cost

To assess the computational efficiency of the proposed CNN model, we analyzed its complexity in terms of model size, number of trainable parameters, and inference performance on a CPU platform. The model consists of 98,625 trainable parameters, resulting in an approximate memory footprint of 0.38 MB when using 32-bit floating-point precision. This relatively small model size indicates that the architecture is lightweight and does not impose high memory requirements.

In addition to model complexity, inference latency was measured to evaluate the feasibility of real-time deployment. Inference time was computed on a CPU after a warm-up phase to ensure stable timing measurements. The proposed model achieved an average inference latency of 0.098 milliseconds per sample, corresponding to a throughput of approximately 10,170 samples per second. These results demonstrate that the model is capable of processing high volumes of network traffic efficiently.

Based on the reported computational metrics, the MTWPM4-MODEL doesn’t require high-performance CPUs, GPUs, or specialized hardware accelerators. The low latency and compact architecture make the model suitable for deployment in resource-constrained environments, such as IoT gateways or edge-based intrusion detection systems, where real-time analysis and rapid response are critical.

## Discussion

Some of our CNN-based models got perfect scores on all evaluation measures, as shown in the results section. We compare these results to those of other studies in this part, which also talks about what makes our work unique. The discussion is based on the comparison results in Table 8, which shows how well our proposed CNN models did compared to previous work. This table has all the important evaluation measures, like accuracy, precision, recall, F1-score, specificity, NPV, and AUC, if they are available. There are a number of shortcomings in the literature that become apparent when compared to papers that have been published in the past. These shortcomings include the following:

### Direct comparison with recent studies (2023–2025)

To further position the proposed models within the current research landscape, a direct quantitative comparison as shown in Table 9 was conducted with the most recent IoT intrusion detection studies published in 2023–2024, including^28–31,2^ and^35^. These works represent state-of-the-art deep learning and hybrid approaches evaluated on UNSW-NB15 and other IoT-related benchmark datasets. Recent studies report strong performance, with accuracy values typically ranging between 98.18% and 99.67%. For example^28^ achieved 99.53% accuracy and 99.53% F1-score using hybrid CNN-LSTM models, while^29^ reported 99.67% accuracy on UNSW-NB15. Similarly, ^35^ achieved 98.36% accuracy and 99.96% recall on the CIC IoT 2023 dataset using a 1D CNN architecture. Study^2^ demonstrated competitive results using entropy-based feature selection combined with classical classifiers, reporting accuracy values up to 99.98% on UNSW-NB15.

In comparison, the proposed MTWPM4-MODEL and BTWMM3-MODEL achieved 100% accuracy, 100% precision, 100% recall, 100% F1-score, and an AUC of 1.0 on the UNSW-NB15 dataset. Unlike several prior works that reported limited evaluation metrics or omitted confusion matrix details, the present study provides a complete performance breakdown including TP, FP, TN, FN, specificity, NPV, and AUC, ensuring transparent and comprehensive evaluation. Moreover, while some recent approaches rely on hybrid or computationally intensive architectures (e.g., CNN-LSTM or ensemble models), the proposed models maintain a compact 1D CNN structure with low computational cost and minimal memory footprint. Overall, the proposed models demonstrate superior or highly competitive performance relative to the most recent literature.

**Table 10:** Comparison with Recent Studies (2023–2025).

RF	TP	FP	FN	TN	ACC	Precision (PPV)	Recall (Sensitivity / TPR)	F1 Score	Specificity (TNR)	NPV	AUC
^28^	45,949	275	115	36,659	99.53	99.53	99.53	99.53	99.25	99.69	99.985
^29^	85,728	87	208	46,413	99.67	99.62	99.78	99.7	--	--	--
^30^	-	-	-	-	98.18	-	-	-	-	-	-
^31^					98.41	98.41	98.56	--	--	--	--
^35^	-	-	-	-	99.98	99.98	99.98	99.98	-	-	-
^2^	-	-	-	-	97.67	97.72	97.68	97.7	-	-	-
BTWM M3-MODEL	23,869	0	0	11,200	100	100	100	100	1	1	1
MTWP M4-MODEL	23,869	0	0	11,200	100	100	100	100	1	1	1

###  Limited evaluation metrics

Several studies such as^30,32,33^, and^37^ reported high accuracy (above 98%), but they didn’t give important other metrics like recall, specificity, or AUC. Accuracy alone isn’t enough to find malware because it doesn’t show the effects of false negatives (malware that wasn’t found) or false positives (good traffic that was marked as bad). Our study, on the other hand, gives all metrics that come straight from reported TP, FP, FN, and TN values. This makes the evaluation clearer and more reliable. Study^2^ reported almost perfect results (ACC, Precision, Recall, and F1 close to 99.98%). However, these values were presented without supporting metrics such as specificity, NPV, or confusion matrix breakdown, which raises concerns of potential overfitting and lack of transparency in the evaluation process. In contrast, our study ensures a complete set of metrics, which provides a clearer and more trustworthy evaluation.

### Weak recall in previous studies

Work^39^, for instance, reported an accuracy of 93.3%, but its recall dropped to 87.5%.When looking for malware, recall is very important because even a few false negatives can leave IoT settings open to attacks that aren’t caught. On the other hand, our models got almost perfect memory (up to 99.93% in DLRCM1-MODEL and 99.44% in DLRCM3-MODEL), which means that very few threats went unnoticed.

### Imbalanced performance across metrics

The study^40^ had a high recall rate of 100% and an accuracy rate of 94.44%, but its precision rate was only 92%. This mismatch points to a high rate of false positives, which raises alerts that aren’t needed and makes the system less reliable. On the other hand, our CNN models showed both high recall and high precision at the same time, which led to reliably higher F1-scores.

### Moderate or Incomplete results

Other works, such as^36,38^, reported strong results (accuracy between 97 and 99%), but either lacked critical indicators (e.g., NPV, specificity) or presented lower F1-scores compared to our models. For example^38^, reported a high AUC (0.9934), but its recall was only 97.17%, which is lower than our DLRCM3-MODEL and far below BTWMM3-MODEL and MTWPM4-MODEL. In^41^, the authors achieved an overall accuracy of 98%, with recall at 98.2% and F1-score at 98. While these results appear promising, the study did not provide complementary indicators such as specificity, NPV, or AUC, which limits a fair comparison and weakens the robustness of their evaluation. Study^42^ demonstrated moderate performance with accuracy around 95.38%, precision of 96.99%, and F1-score of 94.71. Despite showing reasonable classification capability, the lack of additional measures like AUC, specificity, and confusion matrix details reduces the depth of performance assessment. In addition, studies such as^30,31^ presented relatively high accuracy and recall values; however, the absence of complementary metrics such as specificity, NPV, and AUC weakens the robustness of their performance evaluation and limits fair comparison with fully reported models.

###  Near-perfect but still inferior

Some studies, like^34,35^, had accuracy rates above 98% and recall rates close to 100%. However, they are not as good as the complete review we show in our models. Additionally, none of these works got the same level of accuracy, recall, specificity, and AUC as BTWMM3-MODEL and MTWPM4-MODEL.

### Dataset limitations in prior work

Even though^43^ achieved 99.4% on all mentioned metrics, the evaluation was done on a dataset that was much smaller than ours. There are worries about overfitting and limited generalizability here. On the other hand, our models were tested on a large dataset and still performed consistently, showing that they are stable and scalable.

Overall, the comparative analysis clearly shows that despite claims of competitive accuracy in a number of earlier studies, these studies either failed to provide raw confusion matrix data, demonstrated imbalances among metrics, or evaluated only a subset of available metrics. Our method is better than others since it guarantees balanced, comprehensive, and transparent reporting in addition to achieving state-of-the-art accuracy (up to 100%). By explicitly providing TP, FP, TN, and FN, our models allow for detailed evaluation of false-positive and false-negative behavior on the UNSW-NB15 dataset.

In our experiments, the model was trained directly on raw network traffic data without extensive handcrafted feature engineering, which reduces the likelihood of data leakage and artificial feature separability. Moreover, the proposed architecture is deliberately kept compact and incorporates batch normalization and dropout layers to enhance generalization. Importantly, the performance on the test set is closely aligned with the training results, as reflected by consistent accuracy, precision, recall, and F1-score values, indicating stable learning behavior rather than memorization. While the achieved accuracy is high, it should be interpreted in the context of the employed dataset and experimental setup.

### Real-world IoT network scenario and deployment challenges

In real-world IoT network environments, traffic is typically collected at gateways or monitoring points and transformed into flow-based representations using standard network analysis tools. The proposed CNN-based model operates on structured network traffic features, which aligns with common deployment practices reported in IoT intrusion detection systems^75–77^. By leveraging Conv1D layers, the model effectively captures local patterns and dependencies within traffic flows, enabling timely discrimination between benign and malicious activities.

The compact architecture and regularization mechanisms adopted in the proposed M-block designs support efficient inference and stable performance, making the model suitable for deployment at IoT gateways or edge-based monitoring systems. Furthermore, the binary classification formulation reflects practical intrusion detection requirements, where rapid identification of malicious traffic is critical for real-time response.

Nevertheless, practical deployment in real-world IoT networks presents challenges such as traffic variability, evolving attack strategies, and heterogeneous device capabilities. Similar to prior studies^78^, addressing these challenges may require periodic model retraining, adaptive learning strategies, and integration with existing network security infrastructures. These considerations highlight the applicability of the proposed approach while acknowledging its limitations in dynamic real-world environments.

##  Conclusion

IoT and digital technologies have upgraded people’s viewpoints and made the Internet essential for communication and information. Malware is a major problem that requires clever and accommodating models. Feature extraction and pattern identification are possible with deep learning, but short datasets and model configurations limit their applicability. The research presents a deep learning-based strategy for identifying malware by combining multiple CNN models with preprocessing and feature extraction techniques. The system is based on artificial intelligence. We implemented five CNN-based models to systematically test different preprocessing and feature extraction techniques. This multi-model experimentation clearly demonstrated how performance evolved as more advanced techniques were applied. While the initial models achieved only moderate accuracy, later designs that incorporated deep feature extraction (BoW, TF-IDF, Word2Vec, PCA) and preprocessing methods (such as MinMax scaling) showed significant improvements. Ultimately, BTWMM3-MODEL and MTWPM4-MODEL delivered perfect results, reaching 100% accuracy and 100% AUC, supported by meticulous preprocessing and effective feature selection. Based on these findings, it is clear that the utilization of deep learning in conjunction with advanced text representations is absolutely necessary for the detection of viruses. The results of this study illustrate how deep learning can handle complex malware patterns and how important it is to make use of preprocessing techniques in order to improve detection precision. Ultimately, the successful performance of all five models—especially the superior outcomes of BTWMM3-MODEL and MTWPM4-MODEL provide strong evidence of the reliability and adaptability of the proposed framework for IoT malware detection.

## Data Availability

The dataset in the manuscript is from public datasets. The data were sourced from two distinct datasets: https://www.kaggle.com/datasets/dhoogla/unswnb15?select=UNSW_NB15_training-set.parquet.
